# Three-Dimensional SERS Substrates: Architectures, Hot Spot Engineering, and Biosensing Applications

**DOI:** 10.3390/bios15090555

**Published:** 2025-08-22

**Authors:** Xiaofeng Zhou, Siqiao Liu, Hailang Xiang, Xiwang Li, Chunyan Wang, Yu Wu, Gen Li

**Affiliations:** College of Pharmacy and Bioengineering, Chongqing University of Technology, Chongqing 400054, Chinaligen1990@cqut.edu.cn (G.L.)

**Keywords:** SERS, nanowires, dendritic nanostructures, porous frameworks, core–shell nanospheres, biosensing

## Abstract

Three-dimensional (3D) surface-enhanced Raman scattering (SERS) substrates have demonstrated remarkable abilities of ultrasensitive and reproducible molecular detection. The combination of both electromagnetic and chemical enhancement processes, light trapping, and multiple scattering effects of 3D structures are what enhance their performance. The principles of underlying enhancements are summarized systematically, and the main types of 3D substrates—vertically aligned nanowires, dendritic and fractal nanostructures, porous frameworks and aerogels, core–shell and hollow nanospheres, and hierarchical hybrid structures—are categorized in this review. Advances in fabrication techniques, such as template-assisted growth, electrochemical and galvanic deposition, dealloying and freeze-drying, self-assembly, and hybrid integration, are critically evaluated in terms of structural tunability and scalability. Novel developments in the field of biosensing are also highlighted, including non-enzymatic glucose sensing, tumor biomarker sensing, and drug delivery. The remaining limitations, such as low reproducibility, mechanical stability, and substrate standardization, are also noted, and future directions, such as stimuli-responsive designs, multifunctional hybrid platforms, and data-driven optimization strategies of SERS technologies, are also included.

## 1. Introduction

Since its discovery in the 1970s, surface-enhanced Raman scattering (SERS) has become a very effective analytical tool, with the ability to give molecular fingerprint data at extremely high sensitivity. SERS can enhance Raman signals by 10^4^–10^8^ with the use of localized surface plasmon resonance (LSPR) of noble metal nanostructures, making the detection of analytes at the single-molecule level possible in ideal setups [1,2,3]. In comparison with other optical techniques, e.g., fluorescence spectroscopy, SERS has several advantages, including narrow spectral linewidths, low photobleaching, and label-free detection, which make it very desirable in the fields of molecular diagnostics, environmental sensing, food safety, and forensics [4,5].

Traditional SERS substrates are usually two-dimensional (2D) nanomaterials prepared by lithography, colloidal assembly, or self-assembly of noble metals such as Au and Ag. Although these 2D substrates have proven to perform very well under controlled settings, their real-life application is usually limited by the restricted surface area, uneven distribution of hot spots, and low reproducibility, particularly when used in complicated biological matrices like blood plasma or tissue lysates [6,7,8]. In addition, diffusion of the analyte in 2D planes is confined to a limited extent. The planar geometry also limits the number of available hot spots, decreasing the total signal strength and also creating inter-sample variability. In order to address these drawbacks, there has been a growing interest in the creation and synthesis of 3D SERS substrates [9,10,11]. Compared to traditional 2D platforms that are confined to planar geometries with a narrow hot spot distribution, 3D platforms extend the enhancement volume into the third dimension, where hot spots can be packed more densely and longer propagation distances between light and matter can be achieved. As an example, nanopillar arrays, dendritic nanostructures, and porous metal foams have been shown to produce a more isotropic and dense distribution of hot spots than their 2D equivalents, resulting in increased and more consistent SERS signals over a greater sensing area [12].

Recent research has shown the flexibility of 3D SERS substrates in biosensing. As an example, He et al. developed a flexible DNA hydrogel-based SERS-active film capable of conformal detection on irregular or soft surfaces. This 3D structure not only enhances the diffusion efficiency of analytes within the substrate but also significantly increases the signal intensity by boosting the density of hot spots. As a result, it achieves ultrasensitive detection of UO_2_^2+^ with a detection limit as low as 0.838 pM, demonstrating the great potential and practicality of flexible 3D SERS substrates for detecting complex biological samples [13]. In a similar manner, 3D substrates composed of hydrogel with embedded Ag nanoparticles have been applied in dynamic sensing of pH and glucose concentrations in physiological conditions, which provides a responsive SERS performance as a result of their stimuli-responsive character [14,15]. These developments indicate that 3D platforms not only enhance sensitivity and reproducibility but they also have the capability of being integrated with biological systems, and can thus be used in wearable sensors, organ-on-chip systems, and in vivo diagnostic tools. Fabrication-wise, 3D substrates are also able to accommodate a wider variety of architectures, such as hierarchical composites, porous aerogels, and flexible scaffolds. Template-assisted electrodeposition, colloidal self-assembly, freeze-drying, and 3D printing are some of the methods used to fabricate multifaceted nanostructures, with adjustable optical qualities and reproducibility that can be scaled up [16,17,18]. Notably, such methods of fabrication can readily be modified to include molecular recognition features (e.g., antibodies, aptamers), to increase selectivity to specific biomarkers, e.g., cancer proteins, nucleic acids, or pathogens [19].

Considering these breakthroughs, 3D SERS substrates have moved beyond purely academic applications to become biosensing platforms. This review highlights the recent advances in the structure–property relationship of 3D SERS structures—in particular, their improvement mechanisms, fabrication methods, and bioassay analytical capability. In the next sections, we cover the physical principles of SERS enhancements in 3D systems, the main classes of 3D substrates and their fabrication strategies, and biosensing capabilities. In this review, we hope to offer design recommendations and avenues towards creating robust, scalable, and high-performance 3D SERS biosensors.

## 2. Comparison Between 2D and 3D SERS Substrates

To better understand the significance and advantages of three-dimensional (3D) SERS substrates, it is essential to compare them directly with conventional two-dimensional (2D) substrates. While both are designed to enhance Raman signals, their performance, structural attributes, and suitability for biosensing applications differ substantially.

### 2.1. Structural Design and Hot Spot Distribution

Two-dimensional SERS substrates are typically fabricated by depositing metal nanoparticles (e.g., Au, Ag) on flat surfaces through lithography, self-assembly, or drop-casting methods. The enhancement regions, known as “hot spots”, are confined primarily to the surface layer and are often unevenly distributed, which limits the enhancement volume.

In contrast, 3D SERS substrates offer volumetric enhancement due to their extended geometry. Structures such as vertically aligned nanowires, dendritic frameworks, and porous scaffolds create hot spots throughout the vertical and internal volumes, leading to a higher density and more uniform distribution of electromagnetic enhancement regions.

### 2.2. Enhancement of Performance and Reproducibility

The enhancement factors (EFs) of typical 2D substrates range from 10^5^ to 10^7^ under optimized conditions. However, these enhancements often suffer from limited signal uniformity and poor reproducibility due to hot spot heterogeneity and surface roughness variations. On the other hand, 3D SERS substrates can routinely achieve EFs exceeding 10^8^. Their geometries provide robust field confinement and reproducible signal readouts over larger surface areas, which is crucial for practical biosensing applications.

### 2.3. Analyte Accessibility and Compatibility with Complex Matrices

Due to their flat architecture, 2D substrates limit molecular diffusion and interaction with hot spots, particularly in complex or viscous media like blood, saliva, or tissue lysates. Three-dimensional substrates offer porous or vertically structured pathways, facilitate analyte transport and retention, thereby improving signal strength and consistency, even in real biological samples.

### 2.4. Fabrication and Functional Integration

While 2D substrates benefit from well-established lithographic processes, their design flexibility is limited. Three-dimensional SERS substrates, on the other hand, can be fabricated using a range of techniques such as template-assisted deposition, freeze-drying, dealloying, and self-assembly. These approaches allow not only for greater structural tunability but also for the integration of functional elements such as biorecognition molecules, responsive polymers, or photonic layers.

### 2.5. Summary of Comparison

Comparisons of the performance of 2D and 3D substrates are shown in Table 1.

### 2.6. Comparison of Biosensing Applications Between 2D and 3D Substrates

SERS substrates play a critical role in biosensing by enhancing Raman signals of target molecules. Both 2D planar and 3D nanostructured substrates have been widely used, yet they exhibit distinct characteristics affecting biosensing performance.

Two-dimensional SERS substrates, typically composed of flat metal films or patterned nanostructures on planar surfaces, offer relatively simple fabrication and good control over surface chemistry. These substrates enable reproducible measurements due to their uniform analyte distribution on the surface. However, the limited surface area and relatively sparse “hot spots” often result in moderate enhancement factors and lower sensitivity, especially for detecting low-abundance biomolecules in complex samples.

Alternatively, 3D SERS substrates provide a significantly increased surface area and a higher density of electromagnetic “hot spots” through their hierarchical or volumetric nanostructures. This geometry enhances local electromagnetic fields more effectively, leading to stronger Raman signal amplification and ultrasensitive detection capabilities. Additionally, the porous or layered nature of 3D substrates improves molecular capture efficiency by facilitating analyte diffusion and adsorption throughout the volume, which is advantageous for complex biological environments.

Reproducibility, a common challenge in SERS, has been improved in 3D substrates thanks to advances in fabrication techniques such as templating, self-assembly, and 3D printing, enabling consistent nanostructure formation. Nevertheless, 3D substrates typically require more complex and costly fabrication processes compared to 2D substrates, which may impact their scalability and commercial viability.

In summary, while 2D SERS substrates offer an ease of fabrication and controlled surface properties, 3D substrates outperform them in sensitivity, molecular capture, and practical biosensing of complex samples. The choice between 2D and 3D platforms should be based on specific application needs, balancing sensitivity, reproducibility, fabrication complexity, and cost.

## 3. SERS Enhancement Principles in 3D Architectures

In order to derive a comprehensive picture of the performance of 3D SERS substrates, it is imperative to consider each of the three major enhancement mechanisms, namely, electromagnetic (EM) enhancement, chemical enhancement, and multiple scattering effects individually. Each has a different role in signal amplification and is dependent on the structural parameters of the substrate.

### 3.1. Electromagnetic Enhancement

**Figure 1:**
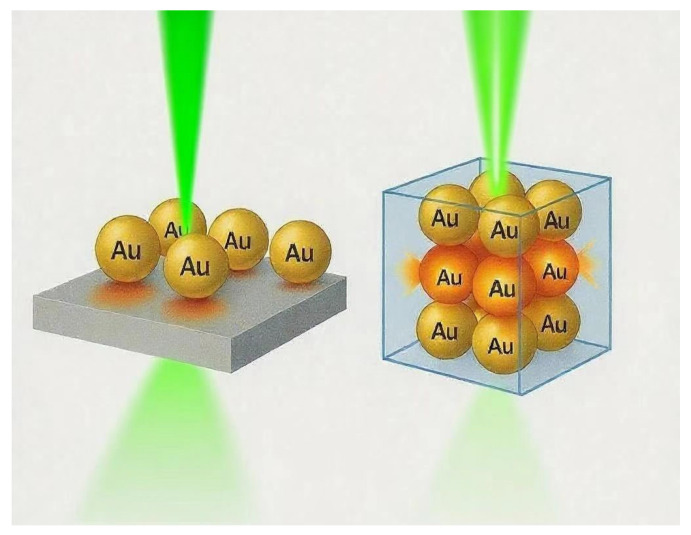
Schematic comparison of EM field distribution between 2D AuNP arrays and 3D AuNP assemblies. © 2025 by the authors.

The most popular mechanism of SERS signal enhancement is the EM mechanism. It arises from localized surface plasmon resonance (LSPR), which refers to the collective oscillation of conduction electrons in nanostructures under resonant illumination at the interface between a conductor and a dielectric. While LSPR is commonly associated with noble metals, it has also been observed in other materials such as post-transition metals (e.g., Al, Bi) and doped semiconductors. By carefully designing nanostructures to support LSPR at the excitation wavelength, intense localized electric fields can be generated, significantly enhancing the Raman scattering cross-section of nearby molecules. The theoretical foundation of this enhancement is based on classical Mie theory and Maxwell’s equations, which describe how nanostructures support LSPR that leads to strong amplification of the local electromagnetic field. The SERS enhancement factor is approximately proportional to the fourth power of the local field amplitude (|E|^4^), where |E| represents the enhanced near-field around the nanostructure, not the incident field itself [20,21,22,23]. Although this mechanism functions in both two-dimensional (2D) and three-dimensional (3D) platforms, 3D architectures offer distinct advantages due to their spatial complexity (Figure 1). The extension of hot spot generation in the vertical (Z) direction allows for a greater density and more uniform distribution of plasmonic coupling regions, vertical field confinement, and the activation of multipolar plasmonic modes, which are difficult to achieve in planar 2D substrates. Finite-difference time-domain (FDTD) modeling of metal–dielectric–metal (MDM) hybrid structures [24] demonstrates intense field localization at tips, junctions, and vertical nanogaps—highlighting the exceptional electromagnetic advantages of 3D geometries.

Several key nanoscale structural parameters—including interparticle gap size, pore size, curvature of features, and surface roughness—play decisive roles in determining the spatial distribution, intensity, and reproducibility of electromagnetic hot spots in 3D SERS substrates. Rather than acting independently, these parameters often work synergistically to localize the EM field and enhance Raman signal output. Among them, nanoscale gaps between metallic nanostructures are the most influential. When the separation between adjacent features is reduced below 5 nm, strong near-field plasmon coupling occurs, leading to orders-of-magnitude enhancement in local field intensity [25]. For instance, Zhang et al. developed a multi-hot spot platform based on nanogap-coupled gold nanoparticles, where the gap between particles is less than 5 nm, leading to strong near-field plasmonic coupling. An experimental SERS enhancement factor as high as 1.36 × 10^8^ was achieved [26]. Lu et al. constructed an atomically defined silver nanocavity and quantitatively measured the upper limit of near-field enhancement in ~1 nm gaps, with electric field enhancement exceeding 1000-fold and a Raman enhancement factor up to 4.27 × 10^10^ with minimal fluctuation [27]. Pore size is equally crucial in porous and aerogel-based architectures, where both analyte accessibility and plasmonic field confinement must be balanced. Nanoporous gold (np-Au), formed via dealloying of Ag-Au alloys, typically presents pore diameters of 10–50 nm, which offer a high surface-to-volume ratio and dense distribution of active sites [28,29]. Such substrates have enabled femtomolar-level detection of analytes like melamine and aflatoxin B1 due to the omnipresence of nanoscale hot spots within the 3D matrix.

The role of feature curvature, especially at sharp tips or branch junctions, is evident in dendritic and fractal nanostructures. These high-curvature regions generate intense field localization via the “lightning-rod effect” [30]. Křápek et al. combined electron energy loss spectroscopy (EELS) experiments with theoretical simulations to quantify the impact of nanosharp tip curvature on local field enhancement. They found that when the tip radius is less than 5 nm, the local electric field enhancement can exceed 10-fold, strongly supporting the intense field localization in dendritic and fractal structures via the lightning-rod effect [31]. Complementing the above, surface roughness—particularly when structured hierarchically at both nano- and microscale levels—further amplifies EM field enhancement. Vertically aligned silicon microcones decorated with Ag nanoparticles exhibit this multiscale roughness, enabling efficient light trapping and synergistic field localization. Chakraborti et al. fabricated vertically aligned silicon nanowire arrays decorated with Ag/Au nanoparticles, forming a hierarchical structure with combined micro- and nanoscale roughness [32]. Using rhodamine 6G (R6G) as a probe molecule, they achieved a SERS enhancement factor as high as 1.5 × 10^7^, with clear spectral signals detectable even at concentrations as low as 10^−9^ M, demonstrating the significant role of multiscale roughness in enhancing local electromagnetic fields and enabling ultrasensitive molecular detection. Collectively, these geometric features contribute to the superior performance of 3D SERS substrates. Their careful engineering—through self-assembly, templating, or top-down lithography—allows not only for ultrahigh sensitivity but also for excellent reproducibility across large sensing areas [33].

Beyond geometric factors, 3D SERS substrates also offer spectral tunability by adjusting nanoparticle shape, size, interparticle distance, and the local dielectric environment. Navarro et al. fabricated hollow octahedral gold nanostructures and achieved tunable LSPR peaks in the range of 530–650 nm by controlling the shell thickness. An enhancement factor as high as 1.1 × 10^8^ was obtained for R6G molecules, demonstrating the significant impact of morphological control on plasmonic resonance tuning [34]. Paganotto et al. systematically studied the effect of nanoparticle shape on plasmonic coupling and found that gold nanocubes formed strong near-field coupling within sub-2 nm wide gaps, achieving a SERS enhancement factor of 2.5 × 10^8^, outperforming spherical and rod-shaped counterparts [35]. Mukherjee et al. developed a 3D substrate based on silica microspheres decorated with silver nanoparticles. By adjusting interparticle spacing and the local dielectric environment, the LSPR peak was shifted from 410 nm to 460 nm, enabling ultrasensitive detection of crystal violet with a detection limit as low as 10^−10^ M [10].

It is worth noting that the enhancement factors (EFs) reported for 3D SERS substrates in this review are frequently in the range of 10^7^ to 10^8^, particularly for well-structured platforms such as dendritic silver networks, nanoporous gold, and multilayered core–shell assemblies. Compared to conventional 2D SERS substrates—such as rough metal films, planar nanoparticle monolayers, or lithographically patterned arrays—where typical EFs range from 10^5^ to 10^7^, the performance of 3D architectures reflects the benefits of volumetric hot spot density, vertical EM field confinement, and a greater analyte-accessible surface area. Although similar EFs can occasionally be achieved with optimized 2D nanoarrays under idealized conditions, these systems often suffer from a limited sensing area, high fabrication cost, and reduced reproducibility. In contrast, 3D substrates offer more consistent and large-area SERS activity, with enhancement factors approaching 10^8^ that are both reproducible and scalable—a feature especially advantageous for biosensing and trace detection applications.

### 3.2. Chemical Enhancement

Although the electromagnetic mechanism plays a key role in the enhancement of signals in SERS, the chemical mechanism (CM) plays a key role in selectivity and specificity particularly where the analyte of interest possesses unique redox or electronic characteristics. Chemical enhancement occurs through photoinduced or ground-state charge transfer between the analyte and the metal surface, which alters the polarizability of the adsorbed molecules and thus alters their Raman scattering cross-section. Its relatively small magnitude (typically 10–10^3^) notwithstanding, CM is also of special significance in the case of engineered 3D substrates where metal–molecule interaction may be both spatially engineered and energetically optimized [36]. The fundamental element of the chemical enhancement mechanism is that of resonance Raman scattering through molecule metal orbital overlap. This may be by photoinduced charge transfer (PICT) or by ground-state electronic interactions, and both are sensitive to the alignment of the molecular energy levels with the Fermi level of the substrate. Perturbation theory based theoretical models have demonstrated that the enhancement of the resonance is dependent on the energy separation between the charge transfer state and the incident photon energy and the electronic state density at the interface [37,38]. The functional domains, the spatial organization, and the controlled porosity in 3D SERS substrates provide the best platform to manipulate such interactions.

**Figure 2:**
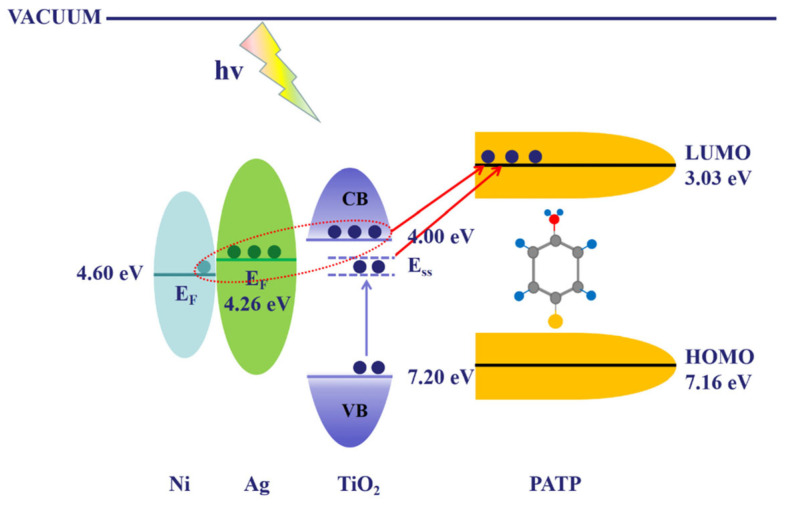
Schematic illustration of CT in PATP/Ag/TiO_2_/Ni system [39]. Reprinted with permission from Wang et al. Copyright 2022 Multidisciplinary Digital Publishing Institute.

The other potential solution is to incorporate semiconductors (such as TiO_2_, ZnO, or MoS_2_) into plasmonic networks to enable directional electron transfer. As an example, Wang et al. systematically investigated the effect of TiO_2_ layer thickness on charge transfer efficiency in Ag/TiO_2_/Ni nanopillar arrays. They found that a TiO_2_ thickness of 10 nm provided an optimal metal–semiconductor–molecule coupling pathway, significantly enhancing the SERS signal. An enhancement factor of approximately 1.2 × 10^7^ was achieved for R6G, clearly demonstrating how tuning semiconductor-layer parameters enables directional electron transfer and improved detection sensitivity [39], as illustrated in Figure 2, which shows a schematic of CT in the PATP/Ag/TiO_2_/Ni system.

In addition to simple metal–semiconductor composites, 3D SERS substrates based on plasmonic metal–organic frameworks (MOFs) represent one of the most promising strategies for enhancing chemical (CM) mechanisms. MOFs possess a high surface area, tunable pore environments, and chemical flexibility, which allow for analyte preconcentration, molecular orientation, and even host–guest charge-transfer resonance. For instance, Liu et al. constructed a plasmonic–MOF hybrid structure by coating gold nanoparticles with a ZIF-8 metal–organic framework, effectively trapping volatile organic compounds (VOCs) within the nanoscale gaps. This design significantly increased the residence time and oriented the arrangement of molecules at plasmonic hot spots, while also inducing host–guest charge-transfer resonance. As a result, SERS detection sensitivity reached the ppb level, fully demonstrating the potential and advantages of MOF materials in enhancing the chemical (CM) mechanism [40]. It has also been shown that the effects of CM can be significantly enhanced through pi-conjugated molecular linkers and functionalized ligands to mediate orbital overlap between the plasmonic metal and the target analytes. Guo et al. constructed a π-conjugated organic semiconductor system and found that molecular orbital delocalization significantly enhanced the charge transfer between the metal and target molecules. Upon interaction with a gold surface, a maximum charge transfer of 0.48 e was observed, accompanied by over a 15-fold increase in SERS signal intensity, highlighting the critical role of π-conjugated systems in enhancing the chemical mechanism [41]. Electrochemical SERS (EC-SERS) experiments also confirm the CM, and the Fermi level of the substrate can also be controlled through applied potential. Guo et al. utilized in situ electrochemical surface-enhanced Raman scattering (EC-SERS) to monitor intermediates during the hydrogen evolution reaction (HER). It was observed that applying a negative potential of −0.5 V (vs. Ag/AgCl) significantly enhanced the Raman signals of adsorbed species such as H_2_O and H*. The SERS intensity of key vibrational modes increased by over eight times compared to the open-circuit potential, confirming that tuning the Fermi level can greatly enhance charge transfer and thus strengthen the chemical mechanism contribution [42]. The other notable example is the 3D porous Ti_3_C_2_-MXene/Au composite, which exhibited strong chemical enhancement for detecting biomolecules. Zhang et al. prepared Au nanoparticle-decorated Ti_3_C_2_ MXene nanoprobes in situ. The unique layered structure and abundant surface functional groups of Ti_3_C_2_ MXene not only enhanced molecular adsorption but also improved interfacial electronic coupling efficiency, further strengthening the chemical enhancement (CM) mechanism. Moreover, the high conductivity of Ti_3_C_2_ MXene effectively reduced carrier recombination, ensuring a high sensitivity and stability of the signals [43].

Additional isolation of CM effects has been performed. As well, time-dependent density functional theory (TD-DFT) calculations have been conducted to correlate molecular orbitals with the experimentally measured Raman band shifts. The molecular selectivity of CM-based enhancement has been demonstrated, where may be selective band intensification via charge-transfer transitions that are resonant in energy, or even vibrational mode suppression, by Birke et al. and Boto et al. [44,45]. CM enhancement is co-expressible with EM effects in a realistic biosensing regime but plays a dominant role when the molecules exhibit a low Raman cross-section or when the analyte is weakly coupled to EM hot spots. It is also the reason why the CM-enhancing 3D platforms may be of special value in the detection of small-molecule biomarkers (e.g., creatinine, uric acid), nucleic acids, or environmental pollutants where specificity is especially important [46,47].

Concisely, 3D SERS structural confinement, electronic band alignment, and interface design are incorporated together along with the integration of chemical enhancement into 3D SERS platforms. In the light of the modern advances in the fabrication methods, there is a better chance to tune these parameters to achieve the analyte-specific, highly sensitive and selective SERS amplification. More overlap of experimental SERS work with quantum-chemical modeling is likely to shed more light on CM in the more realistic 3D systems.

### 3.3. Multiple Scattering and Light Trapping

**Figure 3:**
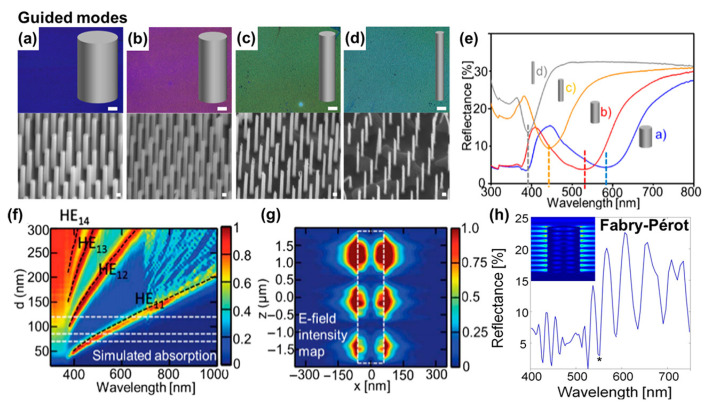
Light trapping and waveguiding effects in VA-SiNW arrays contributing to Raman enhancement. (**a**–**d**) Optical microscopy images (top row; scale bars: 100 μm) and corresponding tilted secondary electron SEM images (bottom row; scale bars: 100 nm) of SiNW arrays with pitch p = 430 nm and length l ≈ 1700 nm: (**a**) d = 118 nm, (**b**) d = 87 nm, (**c**) d = 77 nm, and (**d**) d = 63 nm. (**e**) Reflectance spectra for the arrays in panels (**a**–**d**). (**f**) Contour map of simulated absorption versus wavelength and nanowire diameter (p = 800 nm, l = 3800 nm), with dotted black curves indicating various guided modes. (**g**) Electric field intensity distribution for the HE_11_ leaky mode at *λ* = 670 nm (d = 120 nm), with the white dotted line marking the nanowire boundary. (**h**) Reflectance spectrum of a VA-SiNW array (d = 200 nm, l = 2.4 μm) showing oscillations from Fabry–Pérot resonances. Inset: simulated normalized E-field magnitude at the wavelength marked by an asterisk [51]. Adapted from Bartschmid et al., Copyright 2021, American Chemical Society, with permission.

Raman enhancement in SERS is not limited to the near-field interactions at metal surfaces. Another contributing factor to signal amplification is the influence of structural features on light propagation, scattering, and localization within 3D substrates. Unlike planar surfaces, where incident photons are typically excited during the initial interaction and then undergo surface scattering, 3D structures support more complex optical phenomena—such as multiple scattering, photonic confinement, and waveguiding. These effects collectively increase the effective interaction length between light and analyte molecules, thereby improving excitation efficiency and enhancing the likelihood of detecting Raman-scattered photons [48,49,50]. Another key pathway by which 3D nanostructures amplify light–matter interactions is through photon trapping, where light entering a porous or periodically arranged structure becomes trapped, thereby extending the time it remains within the medium. This prolonged dwell time increases the likelihood of repeated excitation at plasmonic hot spots. Arrays of vertically aligned nanostructures with high aspect ratios—such as silicon nanopillars or nanowires topped with plasmonic nanoparticles—can serve as vertical optical cavities that support guided-mode resonances alongside multipolar plasmon responses. For instance, semiconductor nanowires [51] behave as subwavelength dielectric cylindrical waveguides, channeling and confining light within their cores. This results in enhanced light absorption at specific wavelengths, producing vivid colors in SiNW arrays when the wire diameter d is below 300 nm (Figure 3a–d). Reflectance spectra from such VA-SiNW arrays exhibit distinct dips (Figure 3e), corresponding to the samples shown in panels a–d. Simulated absorption maps (Figure 3f), plotted against diameter and wavelength, reveal that these nanowires sustain hybrid HE1n modes (marked with black dotted curves), where n represents the radial mode number and “1” indicates the azimuthal mode number, describing the electromagnetic field’s radial variation. Increasing the nanowire diameter leads to a redshift of all modes, with thicker nanowires supporting multiple resonances that enhance absorption over a broader wavelength range. The simulated electric field (E-field) distribution for the HE_11_ mode at *λ* = 670 nm (Figure 3g) shows pronounced field enhancement both inside and around the nanowires, indicating leaky waveguiding. Under normal incidence, short-pitch arrangements of nanowires enable coupling between these leaky modes, causing resonance broadening and additional redshifts, while concentrating light within the gaps between nanowires to further boost field intensity. Such near-field coupling has been exploited for improved molecular sensing using SiNWs. Additionally, VA-SiNWs illuminated parallel to their long axis can sustain Fabry–Pérot resonances, in which light reflects between the array’s top and bottom interfaces, forming standing waves that manifest as characteristic dips in the reflectance spectrum. These cavities meet the resonance condition 2L = m*λ*_eff_, where m is an integer and *λ*_eff_ = *λ*_0_/n_eff_, with *λ*_0_ being the vacuum wavelength and n_eff the effective refractive index. For longer nanowires, this condition can be satisfied at multiple wavelengths, yielding oscillatory features in reflectance spectra (Figure 3h). Numerical simulations (inset Figure 3h) clearly display Fabry–Pérot modes as evenly spaced E-field nodes along the nanowire axis, with their spectral position determined by the nanowire length.

More elaborate designs have been made, where vertical scattering, lateral reflection, and refractive index modulation have been designed into multilayered 3D structures [52,53]. As one example, Farid et al. designed a multilayered 3D plasmonic system featuring subwavelength discontinuities [54], enabling light-taming effects through vertical scattering, lateral reflection, and refractive index modulation. This configuration allows incident light to undergo multiple reflections within the structure, forming localized standing waves. As a result, the SERS signal was enhanced by approximately 12-fold, demonstrating the significant role of 3D optical control in strengthening light–matter interactions. The contribution of optical trapping can also be varied by varying the geometric parameters, such as pore size, pillar height, lattice spacing, and filling fraction. It is also interesting to add that the light trapping reduces the backscattering losses and enhances the collection efficiency in SERS applications using portable instruments [55]. The angular scattering of the scattered light in the 3D structures is characterized in a way that promotes forward scattering and diffusive propagation, which promotes the congruence with the angles of collection of objective lenses. Practically, it implies more stable, stronger signals and no necessity of applying brighter lasers or longer acquisition durations [56]. The combination of such optical effects is particularly applicable for detecting analytes in low-concentration or turbid media. As a case in point, light attenuation and random scattering in biological fluids are major challenges to conventional SERS. However, 3D substrates that enable light recycling internally, i.e., SERS-active fiber mats or porous hydrogels, have shown the ability to overcome such impediments [57]. As SERS is being translated into in vivo and real-time applications, the design of 3D photonic–plasmonic hybrid structures will become even more important. The synergy of nanophotonic and plasmonic principles in designs will enable not merely enhanced sensitivity but also spectral selectivity, normalization, and spatial resolution in sensing, which is of paramount importance in clinical and environmental applications.

However, despite significant progress in understanding the mechanisms of SERS, several key questions remain. In particular, the individual contributions of EM and chemical (CM) enhancement mechanisms are difficult to quantify in complex 3D systems, due to their similar spectral signatures and spatial heterogeneity. These mechanisms require standardized isolation procedures in comparative studies and benchmarking of substrates. Future studies must be aimed at the accurate design of multifunctional SERS substrates combining EM-optimized shapes and CM-active surfaces. Real-time characterization methods are also possible (including tip-enhanced Raman spectroscopy (TERS) and ultrafast spectroscopy) and can provide time-resolved enhancement. In addition, the new possibilities of structure–property relationships’ comprehension and prediction in SERS-active systems are presented by simulations performed with the use of machine learning. It is possible to push the boundaries of SERS sensitivity, reproducibility, and molecular specificity by a combination of both EM and CM effects in full, and by rational 3D structural design.

## 4. Types of 3D SERS Substrates

The SERS activity of 3D substrates is highly sensitive to their structural design, which determines the formation, distribution, and stability of EM hot spots. In the following section, we describe five major categories of 3D SERS architectures, focusing on their morphological characteristics, enhancement mechanisms, fabrication feasibility, and application relevance.

### 4.1. Vertically Aligned Nanowire and Nanorod Arrays

Among 3D SERS geometries, vertically aligned nanowire and nanorod arrays are perhaps the most extensively studied. These structures support directional plasmonic coupling and are compatible with scalable fabrication techniques. They are typically fabricated using methods such as anodized aluminum oxide (AAO) templating, glancing angle deposition (GLAD), or hydrothermal growth.

**Figure 4:**
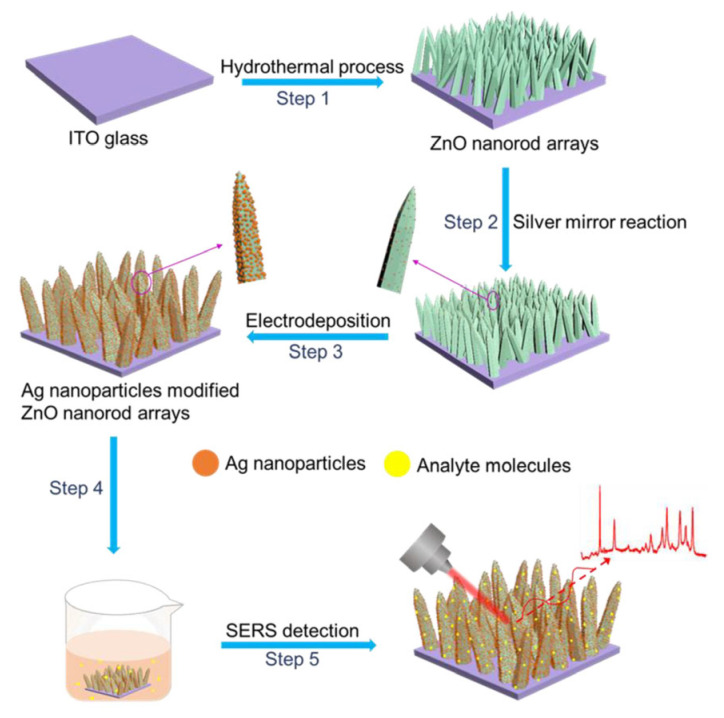
Fabrication process and plasmonic enhancement mechanism of Ag/ZnO nanorod arrays for SERS detection. Step 1: Uniform ZnO nanorod arrays are synthesized on ITO glass via a hydrothermal method. Step 2: Silver nuclei are deposited onto the ZnO nanorod surfaces using a silver mirror reaction. Step 3: These nuclei grow into larger Ag nanoparticles through electrodeposition. Step 4: The substrate is immersed in the analyte solution to adsorb target molecules. Step 5: The Ag/ZnO nanorod arrays function as SERS-active substrates for ultrasensitive detection of organic compounds [73]. Reprinted with permission from Bartschmid et al. Copyright 2021 Elsevier BV.

The large aspect ratio of vertically aligned rods enables efficient axial confinement of the electromagnetic field, resulting in the formation of vertically distributed SERS hot spots [58,59,60,61,62,63,64,65,66,67,68,69,70,71,72]. As an example, Li et al. fabricated Ag/ZnO nanorod arrays via a hydrothermal growth process, followed by surface decoration with silver nanoparticles [73] (schematic in Figure 4). Incorporating Ag nanoparticles onto the tall ZnO nanorods generated densely packed SERS active sites and strong electromagnetic localization, achieving an enhancement factor as high as 2.7 × 10^8^. These results highlight the superior performance of vertically oriented, three-dimensional nanostructures in SERS-based sensing.

Furthermore, the nanowire scaffolds can be chemically enhanced by incorporating materials such as ZnO or TiO_2_, which provide additional charge-transfer pathways [74,75]. These structures also serve as heterogeneous optical cavities that prolong the photon lifetime and promote multiple scattering. Collectively, these attributes make vertically aligned arrays particularly well-suited for analyte pre-concentration and reproducible SERS mapping over large surface areas.

Such arrays are generally fabricated using bottom-up, template-assisted methods, including AAO templating, glancing angle deposition, and hydrothermal synthesis. These approaches allow precise control over nanowire height, spacing, and alignment. Additional details regarding synthesis parameters and scalability are discussed in Section 5.1.

### 4.2. Dendritic Nanostructures

Dendritic nanostructures are characterized by their self-similar, fractal-like, and multibranched geometry. This unique topology, featuring dense nanoscale protrusions and narrow interbranch gaps, is highly conducive to broadband plasmon resonance and strong electromagnetic field localization [76,77,78,79,80]. Fractal 3D gold nanodendrites synthesized via templated galvanic displacement reactions exhibit numerous sharp edges and tips, forming ultra-dense “lightning rod” hot spots. Due to their high specific surface area and densely packed SERS active sites, these nanodendrites show great potential for applications in both chemical sensing and catalysis. Their hierarchical structure offers multifunctionality, as each generation of branching arms acts as a nanoantenna, promoting strong interbranch plasmon coupling and resulting in the formation of spatially distributed electromagnetic hot spots [81]. Notably, the inherent surface roughness of dendritic structures allows SERS signal enhancement over a broad wavelength range. Fractal SERS substrates synthesized by diffusion-limited aggregation (DLA) or aggregation-induced self-assembly (AISA) can form high-density plasmonic junctions with interparticle gaps of less than 2 nm.

For example, Zhang et al. developed a hierarchical SERS substrate that combined an ordered silicon micropyramid array with uniformly distributed silver nanoparticles. The micropyramids facilitated light trapping and multiple reflections, while the AgNPs generated abundant plasmonic hot spots. This synergistic structure enabled ultrasensitive detection of Rhodamine 6G down to 10^−12^ M with excellent signal uniformity and reproducibility, demonstrating the effectiveness of engineered topologies for enhancing plasmon resonance and field localization [82].

Dendritic nanostructures are typically fabricated via electrochemical deposition, gas–liquid interfacial reactions, or galvanic replacement methods. These approaches are generally rapid and template-free. The growth dynamics and branching control mechanisms of dendritic morphologies are discussed in detail in Section 5.2.

### 4.3. Porous Frameworks and Aerogel-Based Scaffolds

Porous frameworks, including metal–organic frameworks (MOFs), covalent organic frameworks (COFs), and mesoporous silica materials, have been extensively explored as 3D SERS substrates due to their tunable porosity, high surface area, and ability to host plasmonic nanoparticles [83,84,85,86].

**Figure 5:**
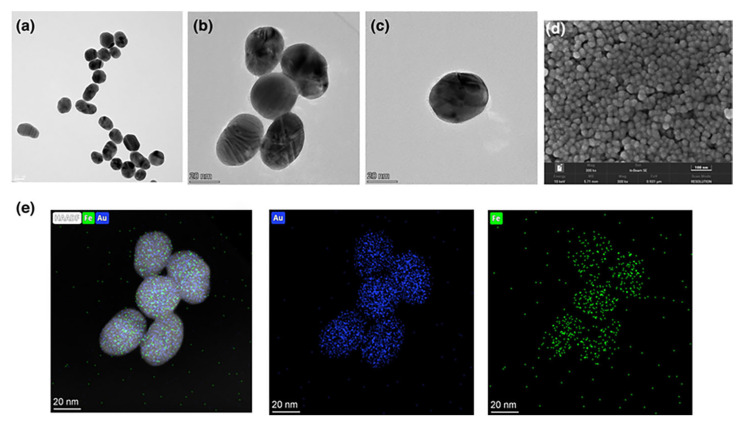
Structural characterization of Au@MIL-101 hybrid nanostructures for enhanced SERS performance. (**a**) Transmission electron microscopy (TEM) image of Au nanoparticles (AuNPs). (**b**,**c**) TEM images showing Au@MIL-101 composites. (**d**) Scanning electron microscopy (SEM) image of Au@MIL-101 integrated with PMMA/DT. (**e**) Scanning TEM (STEM) combined with energy-dispersive spectrometry (EDS) elemental mapping of Au@MIL-101 [88]. Reproduced with permission from Xue et al. Copyright 2024 Royal Society of Chemistry.

MOFs, composed of metal ions coordinated with organic ligands, exhibit diverse architectures with adjustable pore sizes and chemical environments. This structural flexibility facilitates the uniform integration of plasmonic nanoparticles (e.g., Au, Ag, Cu) into their frameworks, forming hybrid composites that combine EM and CM [87]. The metal chains in MOFs facilitate electron transport, while their porous interconnected networks enhance analyte diffusion and enlarge the effective interaction surface with plasmonic sites. For instance, Chen et al. developed a MOF-functionalized gold nanoparticle array (AuNPs@MIL-101), where MIL-101 was assembled onto AuNPs, creating an ordered hybrid architecture (illustrated in Figure 5) [88]. This design greatly increased hot spot density and molecular adsorption capacity, achieving paraquat detection in cereals down to 7.1 × 10^−9^ M. These findings demonstrate MOFs’ crucial dual function in nanoparticle stabilization and enhanced SERS activity.

COFs, with extended π-conjugated structures, also provide favorable environments for charge-transfer interactions with analytes, complementing the EM enhancement of embedded metal nanoparticles. Yang et al. [89] synthesized AgNP-decorated COFs that achieved ultrasensitive detection of food pollutants at picomolar concentrations. The porous structure of COFs facilitated rapid analyte diffusion, ensuring fast and efficient sensing.

**Figure 6:**
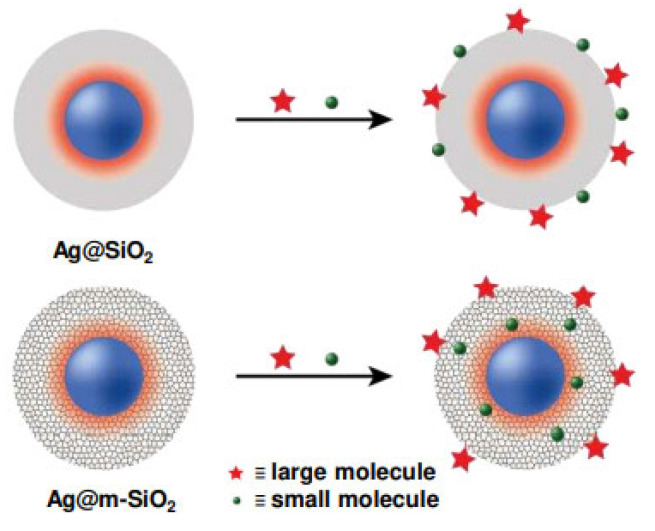
Diagram illustrating silver nanoparticles capped with microporous silica (Ag@SiO_2_), which blocks the passage of molecules (top panel), compared to silver nanoparticles coated with mesoporous silica (Ag@m-SiO_2_), allowing selective sieving of large molecules while enabling SERS detection of smaller molecules (bottom panel). The blue sphere and surrounding porous network depict the Ag nanoparticle core and the mesoporous silica (m-SiO_2_) shell, respectively, where ‘m’ denotes mesoporous. The orange–red halo represents the electric field generated by the Ag core. Larger molecules (red stars) are excluded by the shell, while smaller molecules (green spheres) permeate the pores of m-SiO_2_ and are exposed to the enhanced electric field, boosting their Raman signal [95]. Reproduced with permission from Sharma et al. Copyright 2025 Royal Society of Chemistry.

Mesoporous silica-based aerogels and xerogels serve as robust, low-density 3D scaffolds for SERS applications. These can be functionalized with plasmonic nanoparticles to create highly porous and interconnected networks [90,91,92,93,94]. Singh et al. developed core–shell Ag@m-SiO_2_ particles by coating plasmonic silver nanoparticles with a mesoporous silica shell, forming a structure capable of both signal enhancement and molecular sieving [95]. As schematically illustrated in Figure 6, the mesoporous shell permits small target molecules to diffuse through and interact with the enhanced electromagnetic field near the Ag core (indicated by the orange–red halo), while excluding larger interfering species. This dual function enabled sensitive and selective SERS detection, demonstrating excellent reproducibility and practical applicability.

In addition, polymeric foams and noble-metal-decorated aerogels serve as lightweight and flexible SERS supports. Fu et al. [96] fabricated Ag-TiO_2_hybrid aerogels with hierarchical porosity and optical transparency, enabling real-time monitoring of environmental pollutants. Moreover, incorporating stimuli-responsive hydrogels into SERS platforms introduces tunability. These hydrogels respond to environmental changes—such as pH, temperature, or ionic strength—by swelling or shrinking, which dynamically adjusts nanoparticle spacing and the local refractive index. This modulation allows real-time tuning of plasmonic coupling and SERS intensity, improving reproducibility and enabling multiplexed or adaptive sensing systems [97,98].

The high light-scattering properties of these porous materials increase photon–analyte interaction times, further enhancing Raman signal amplification. Common fabrication strategies include metallic dealloying, sol–gel synthesis, and freeze-drying techniques, which are discussed in detail in Section 5.3.

### 4.4. Core–Shell and Hollow Nanosphere Assemblies

**Figure 7:**
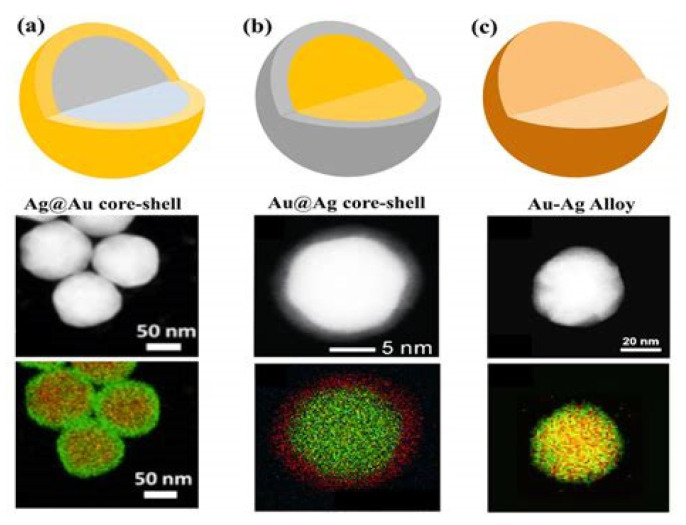
Illustration of spherical bimetallic Au–Ag nanoparticles as (**a**) Ag@Au core–shell structures, (**b**) Au@Ag core–shell structures, and (**c**) alloy nanoparticles, along with their respective TEM images and EDX elemental maps [99]. Reproduced with permission from Li et al. Copyright 2017 Elsevier.

Core–shell nanoparticles, such as Au@Ag and SiO_2_@Au, are among the most widely utilized structures for SERS enhancement due to their tunable plasmonic interactions and strong electromagnetic field confinement at the core–shell interface, as demonstrated by the TEM images and EDX mappings of spherical bimetallic Au–Ag nanoparticles presented in Figure 7 [99,100]. By adjusting the dielectric environment and the thickness of the shell, the LSPR can be tailored to match specific excitation wavelengths, thereby optimizing the Raman signal. These nanoparticles can be assembled into ordered 3D films using methods such as layer-by-layer deposition, drop-casting, or the Langmuir–Blodgett technique. Such structured assemblies improve the reproducibility and uniformity of hot spot distribution, which is essential for quantitative SERS analysis.

Hollow nanostructures—such as gold nanocages and nanoshells—offer additional advantages due to their internal cavities. These structures support dual-mode enhancement, with electromagnetic hot spots formed on both the inner and outer surfaces, and provide tunable scattering profiles based on shell thickness and geometry. The presence of cavity modes further increases the optical path length, enhancing light–matter interactions.

For example, Song et al. [83] fabricated multilayered films of Au nanospheres on SiO_2_ substrates with precisely controlled interparticle distances. These structures exhibited enhancement factors exceeding 10^7^ and relative standard deviations (RSDs) below 5%, making them highly suitable for biosensing applications that require uniform signal distribution, high optical transparency, and low background noise.

Common fabrication techniques for core–shell and hollow nanospheres include seed-mediated growth, shell coating, and self-assembly, which are discussed in detail in Section 5.4.

### 4.5. Hierarchical Hybrid Architectures

**Figure 8:**
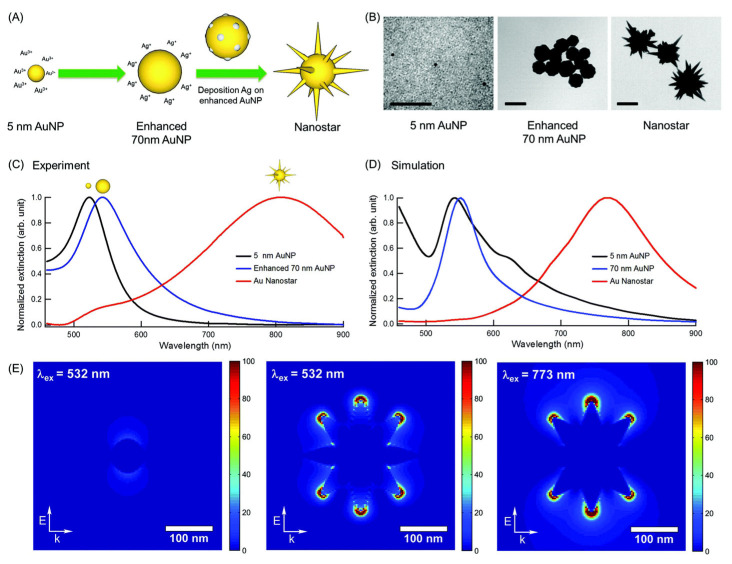
Synthesis, plasmonic properties, and tip-induced electromagnetic field enhancement of Au nanostars relevant to single-molecule SERS detection. (**A**) Schematic representation of the synthesis process for gold nanostars. (**B**) Transmission electron microscopy (TEM) images showing 5 nm Au nanoparticles (AuNPs), enlarged 70 nm AuNPs, and gold nanostars (scale bar: 100 nm). (**C**) Extinction spectra for 5 nm AuNPs (black), 70 nm AuNPs (blue), and nanostars (red), with peak absorbances at 519 nm, 543 nm, and 809 nm, respectively. (**D**) Finite-difference time-domain (FDTD) simulated scattering spectra of the corresponding nanoparticles. (**E**) Simulated local electric field distributions around a 70 nm spherical AuNP at *λ* = 532 nm, an Au nanostar at *λ* = 532 nm, and an Au nanostar at *λ* = 773 nm. The color scale represents intensity in |*E*|^2^. Arrows labeled *E* and *k* indicate the polarization and propagation directions of the incident light [101]. Reproduced with permission from Song et al. Copyright 2023 Multidisciplinary Digital Publishing Institute.

Hierarchical hybrid structures combine diverse architectural elements across multiple scales to realize synergistic improvements in SERS efficiency. For example, star-shaped nanoparticles can be incorporated into nanowire frameworks to merge the intense field enhancement at nanostar tips with vertical electromagnetic coupling along the nanowires. Likewise, embedding nanoporous architectures within hydrogel matrices provides both a high density of hot spots and environmental sensitivity. Tatar et al. reported the self-assembly of gold nanostars into thin films stabilized by polyvinylpyrrolidone (PVP), enabling single-molecule detection of crystal violet through plasmonic enhancement at the nanostar tips [101]. The junctions between the arms, characterized by sharp curvatures, serve as highly localized electromagnetic hot spots (Figure 8).

Another compelling example is the design of gradient hydrogel composites containing AuNPs. These materials utilize a gradient refractive index to promote light trapping and feature a soft, deformable matrix that facilitates analyte diffusion and responsive structural changes. Such properties make them especially attractive for wearable or in situ diagnostic applications, where mechanical flexibility and signal robustness are critical. The fabrication of these hybrid architectures typically involves multistep or multimodal approaches, combining techniques such as self-assembly, crosslinking, template etching, and nanoparticle embedding. Section 5.4 discusses these integrated fabrication strategies in greater detail.

A recent trend in 3D SERS substrate development involves hybrid photonic crystal–hydrogel scaffolds, which merge the optical resonances of photonic crystals with the mass transport and tunability of hydrogels [102]. These composites simultaneously optimize electromagnetic field enhancement—via photonic band gap effects—and provide a hydrated, biocompatible environment that facilitates analyte adsorption and detection. Incorporating plasmonic nanoparticles within these hybrid scaffolds results in synergistic effects, with enhancement factors (EFs) exceeding 10^9^ and excellent signal homogeneity, making them well-suited for ultrasensitive detection of environmental pollutants and biomolecules [103,104]. The optical and structural characteristics of these 3D photonic-hydrogel substrates allow for extraordinary Raman signal amplification. Their tunability enables precise control over light–matter interactions, while their high analyte accessibility and environmental stability make them highly promising for biomedical diagnostics, environmental monitoring, and chemical sensing.

By leveraging multiscale architecture, hierarchical nanostructures create synergistic effects at both the nano- and microscales. The ability to finely tune their geometric design allows for the formation of densely packed and uniformly distributed hot spots, significantly enhancing local electromagnetic fields. Additionally, the high specific surface area and interconnected porosity of aerogels and porous frameworks facilitate efficient analyte diffusion and hot spot formation, thereby balancing enhancement efficiency with detection sensitivity.

Functional substrates such as photonic crystals and hydrogel scaffolds, when combined with optical resonance tuning and the mechanical flexibility of soft materials, not only support substantial electromagnetic enhancement but also provide environmental compatibility and dynamic tunability—key advantages for biomolecular sensing platforms.

In summary, the emerging field of 3D SERS substrates leverages a diverse array of nanostructural designs to enhance the density and uniformity of electromagnetic hot spots, thereby significantly improving signal intensity, sensitivity, and detection reproducibility in Raman spectroscopy. Each type of 3D SERS substrate discussed in this review offers distinct and complementary advantages. To provide a clear comparison of the various 3D SERS architectures discussed in this review, Table 2 summarizes their key structural features, enhancement factors, reproducibility, practical advantages, and limitations in biological applications. This comparative overview serves as a practical guide for selecting appropriate SERS substrate types based on application-specific requirements. This comparative overview serves as a practical reference for selecting the most suitable SERS substrate based on specific application requirements. Moving forward, the development of composite materials and structural optimization will be essential to achieving high sensitivity, signal uniformity, and multifunctionality, thus accelerating the widespread adoption of SERS technology in environmental monitoring, medical diagnostics, and food safety. Moreover, the integration of computational modeling with experimental characterization techniques will provide deeper insights into electromagnetic enhancement mechanisms, forming a solid theoretical foundation for the rational design of high-performance 3D SERS substrates and their practical implementation.

## 5. Fabrication Strategies

Fabrication techniques are critical in determining the structural properties, reproducibility, and performance of 3D SERS substrates. Among the most widely used approaches are template-assisted growth, self-assembly, and etching, owing to their versatility and ability to offer precise morphological control. These methods enable the construction of complex 3D architectures with tailored plasmonic functionalities and consistent hot spot distributions, essential for sensitive and reproducible SERS detection.

### 5.1. Template-Assisted Growth

Template-assisted growth utilizes pre-patterned templates to direct the nucleation and assembly of plasmonic nanostructures into highly ordered 3D configurations. The periodicity and tunable dimensions provided by templates are major advantages, allowing for the reliable production of substrates with abundant and uniformly distributed hot spots.

**Figure 9:**
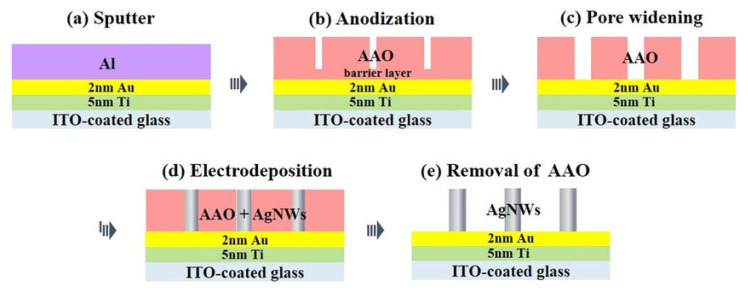
Schematic illustration of the fabrication process for AAO-template-assisted, vertically aligned metallic nanowire arrays, designed to provide uniform electromagnetic field enhancement and reproducible SERS substrates. The process includes: (**a**) sputtering Ti/Au/Al layers on ITO-coated glass; (**b**) anodization of the Al layer to form AAO with a barrier layer; (**c**) pore widening treatment of AAO; (**d**) electrodeposition of metallic nanowires (e.g., Ag) into the AAO nanopores; and (**e**) removal of the AAO template to yield free-standing, vertically aligned nanowire arrays [106]. Reproduced with permission from Mo et al. Copyright 2015 Springer-Verlag.

Anodic aluminum oxide (AAO) is among the most frequently utilized templates due to its precisely ordered nanoporous arrays with tunable pore sizes and spacing. Vertically aligned metallic nanowires or nanotubes can be fabricated within AAO templates by methods such as electrochemical deposition or galvanic replacement, producing large-area substrates with consistent electromagnetic field enhancement throughout the array [105,106,107] (refer to Figure 9). For example, Du et al. demonstrated that Ag nanowire arrays fabricated using AAO templates achieved uniform interwire spacing (~30 nm), which produced highly reproducible SERS signals with relative standard deviations (RSDs) of less than 7% across centimeter-scale areas [108]. This high reproducibility stems from the precise spatial localization and orientation enabled by the template.

Similarly, inverse opal structures have been fabricated using colloidal crystal templates such as polystyrene (PS) or silica microsphere arrays. These inverse opals, due to their periodic lattice voids, can support photonic band gap effects and enhance light–matter interactions, further boosting SERS sensitivity [109].

In addition, biologically derived hierarchical structures—such as diatom frustules and butterfly wings—have attracted attention as natural 3D SERS templates. Their inherently high specific surface area and complex micro/nanostructured features provide numerous scattering centers and sites for hot spot generation. For instance, Li et al. deposited silver onto diatom frustules, yielding high-density plasmonic hot spots with enhancement factors exceeding 10^8^ and excellent signal reproducibility [110].

Template-assisted methods also facilitate the construction of multi-material and core–shell heterostructures. Through sequential deposition within templates, complex architectures combining plasmonic metals and functional coatings can be realized. These hybrid structures offer enhanced stability, selectivity, and performance in biosensing applications [111,112].

However, challenges remain in terms of template removal and scalability. Future research should aim to develop efficient and reusable template systems with simplified dissolution processes to further advance the practical application of this technique.

### 5.2. Electrochemical and Galvanic Formation of Dendritic Structures

**Figure 10:**
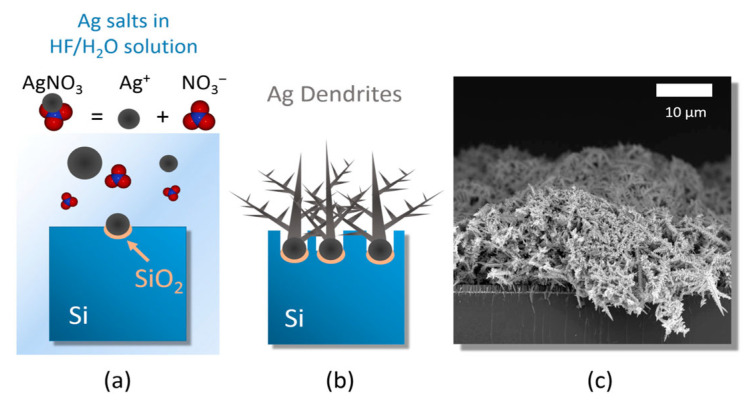
Electrochemical synthesis and structural features of Ag dendritic nanostructures for broadband SERS enhancement. (**a**) Schematic illustrating the growth of silver dendrites initiated by the dissolution of AgNO3 salts in an HF/H_2_O solvent, resulting in silver nanoparticle formation and their deposition onto silicon substrates. (**b**) Further growth of silver dendrites occurs through additional Ag^+^ ion incorporation onto initial seed particles. (**c**) Cross-sectional scanning electron microscopy (SEM) image showing a dense carpet of silver dendrites deposited on silicon bulk [113]. Reproduced with permission from Kenneth et al. Copyright 2020 American Chemical Society.

A unique category of three-dimensional SERS substrates includes dendritic and fractal nanostructures fabricated through electrochemical or galvanic synthesis. These architectures exhibit hierarchical, multibranched shapes with abundant nanoscale gaps, delivering broadband plasmonic responses. Unlike template-assisted techniques, these methods depend on self-assembly and diffusion-limited growth, which naturally produce complex multiscale structures without requiring predefined templates or molds [113] (see Figure 10).

The fundamental mechanism of electrochemical dendrite formation is governed by non-equilibrium ion transport and field-concentrated tip growth. Under conditions of high cathodic overpotential and localized electric fields, metal ions (typically Ag^+^ or Au^3+^) are preferentially reduced and deposited at the tips of existing structures. This process promotes self-similar branching growth, forming high-curvature regions ideal for SERS hot spots. The morphology of the dendritic structures can be finely tuned by adjusting parameters such as electrolyte composition, ion concentration, deposition time, and substrate conductivity.

High-density interbranch junctions with sub-10 nm gaps are often produced through diffusion-limited aggregation (DLA), resulting in fractal-like assemblies. These architectures offer ample surface area and spatially distributed plasmonic interactions, and they are particularly suited for broadband or non-resonant SERS enhancement. Moreover, the inherently irregular and rugged geometry enhances light trapping and multi-angle scattering, contributing to signal amplification.

Electrochemically fabricated dendritic substrates have shown an outstanding SERS performance. For instance, Ye et al. [114] demonstrated Ag dendrites prepared via controlled-potential deposition, featuring branch tips with radii less than 10 nm and enhancement factors exceeding 10^8^ for the trace detection of rhodamine 6G. Similarly, Bai et al. [115] utilized DLA to produce large-area Ag fractal films with a hot spot coverage over 90%, enabling centimeter-scale SERS mapping.

Despite their excellent plasmonic properties and scalability, electrochemical and interfacial growth techniques face challenges in morphological reproducibility. The stochastic nature of dendritic growth leads to significant batch-to-batch variations in branch density, interstitial gap size, and surface roughness [116]. Additionally, these nanostructures tend to be mechanically fragile and are not ideal for applications requiring flexible or transferable substrates.

To overcome these limitations, recent studies have explored hybridizing dendritic structures with polymers, oxides, or self-assembled monolayers to enhance their environmental and mechanical stability. Particularly promising is the integration of electrochemical growth with soft lithographic patterning or masking techniques, which can introduce a level of spatial ordering while retaining the benefits of stochastic growth. This hybrid approach offers a cost-effective, template-free, and scalable method to fabricate hierarchically organized nanostructures tailored for high-performance SERS applications.

### 5.3. Dealloying and Freeze-Drying Strategies for Porous and Aerogel Frameworks

Aerogel-based scaffolds and porous metallic frameworks represent a prominent class of 3D SERS substrates, known for their high surface area, multidirectional light scattering, and efficient analyte diffusion. These substrates are typically fabricated through two major approaches: dealloying of bimetallic systems and sol–gel-based freeze-drying. Both strategies allow the generation of volumetric plasmonic nanostructures without the need for external patterning or lithographic techniques.

**Figure 11:**
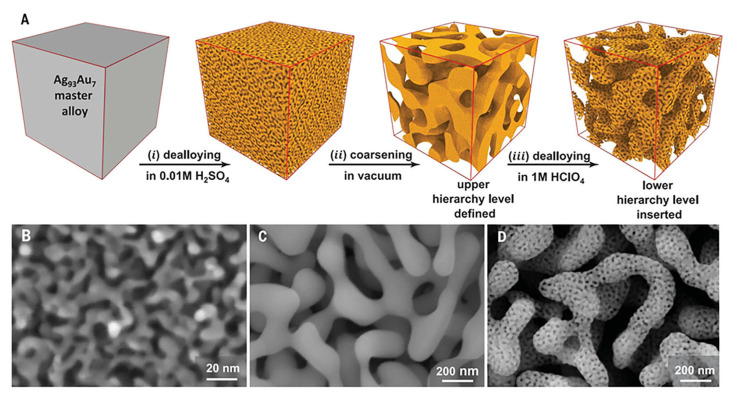
Fabrication of hierarchical nanoporous gold via sequential dealloying. (**A**) Schematic overview of the process steps: (i) initial dealloying of an Ag-rich precursor alloy creates a nanoporous network containing a high concentration of residual silver; (ii) thermal annealing induces coarsening of the ligament framework; (iii) a second dealloying step produces a finer nanoporous microstructure at a lower hierarchical level. (**B**–**D**) Scanning electron microscopy (SEM) images showing the microstructure after the first dealloying (**B**), coarsening (**C**), and second dealloying (**D**) stages [118]. Reproduced with permission from Liu et al. Copyright 2023 American Chemical Society.

The dealloying process involves the selective removal of a less noble metal (e.g., Ag) from a chemically mixed alloy (e.g., Ag-Au), typically through immersion in nitric acid or other chemical etchants [117]. The driving force of this process lies in the difference in standard electrode potentials between the constituent metals. During the preferential dissolution of Ag atoms, the remaining Au atoms undergo surface rearrangement, forming a bicontinuous network of nanoscale ligaments and pores. This hierarchical nanostructure formation is illustrated in Figure 11, which shows the stepwise evolution of the nanoporous morphology through successive dealloying and annealing processes [118]. The resulting nanoporous gold (np-Au) structures exhibit high curvature, roughened surfaces, and interconnected porosity, all of which contribute to strong local plasmonic resonances and a high density of SERS active sites.

**Figure 12:**
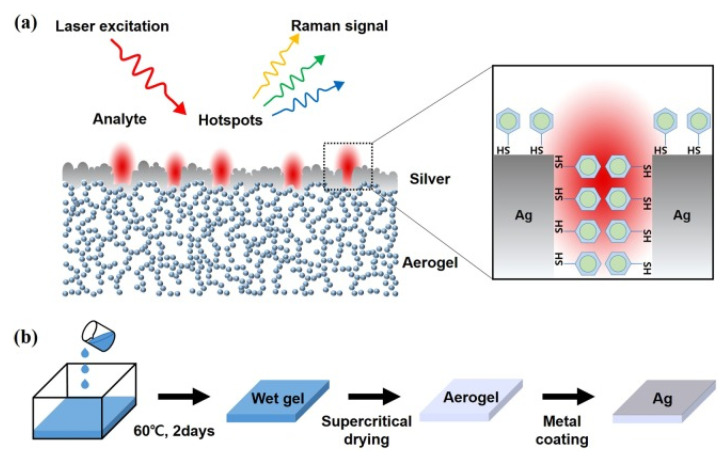
(**a**) Schematic of plasmon-enhanced Raman scattering on silver-coated silica aerogel, highlighting light–matter interaction within the porous matrix. (**b**) Fabrication process of the aerogel-based SERS substrate: silica wet gel was synthesized using MTMS, surfactant, and acetic acid, followed by supercritical CO_2_ drying (80 °C, 13.5 MPa) to preserve porosity. A silver layer was deposited via electron beam evaporation to form plasmonic nanogaps. Reprinted with permission from Shi et al. [119]. Copyright 2021 American Association for the Advancement of Science.

In parallel, aerogel structures are typically synthesized through sol–gel chemistry, followed by freeze-drying or supercritical drying to preserve their porous architecture [119] (see Figure 12). In this method, plasmonic nanoparticles (such as Ag, Au, or hybrids like Ag-TiO_2_) are incorporated into a polymeric or inorganic gel matrix. The solvent is subsequently removed under conditions that prevent capillary collapse, thereby maintaining the intrinsic mesoporous or macroporous structure. The resulting aerogels exhibit an ultralow density, high internal surface area (often >500 m^2^/g), and excellent optical transparency. These properties facilitate deep light penetration, improved analyte access, and enhanced in situ detection capabilities.

Despite the clear structural advantages of these approaches, they also present certain challenges. The dealloying process, although chemically simple, tends to be waste-intensive, often generating significant amounts of metal ion effluents and requiring the use of corrosive acids. Moreover, the tunability of the resulting porous structures is limited by the original microstructure of the alloy, which largely predetermines the pore size distribution and structural uniformity [120]. In contrast, freeze-dried aerogels are more environmentally friendly but inherently mechanically fragile, which limits their use in flexible or field-deployable devices [121]. Another technical challenge lies in achieving a uniform dispersion of plasmonic nanoparticles within the aerogel matrix. At higher loading concentrations, nanoparticle aggregation becomes more likely, which can reduce the performance and consistency of the substrate. To address these issues, researchers have explored greener dealloying alternatives, such as electrochemical dealloying, which reduces chemical waste and improves structural control. Likewise, the mechanical integrity and reproducibility of aerogels can be enhanced through composite reinforcement techniques, including polymer crosslinking and the incorporation of oxide-based frameworks. Importantly, the porous nature of both dealloyed and aerogel-based substrates is particularly well-suited for applications that require rapid analyte diffusion, low background interference, and real-time sensing of gases or volatile compounds.

### 5.4. Self-Assembly of Core–Shell and Hollow Nanostructures

**Figure 13:**
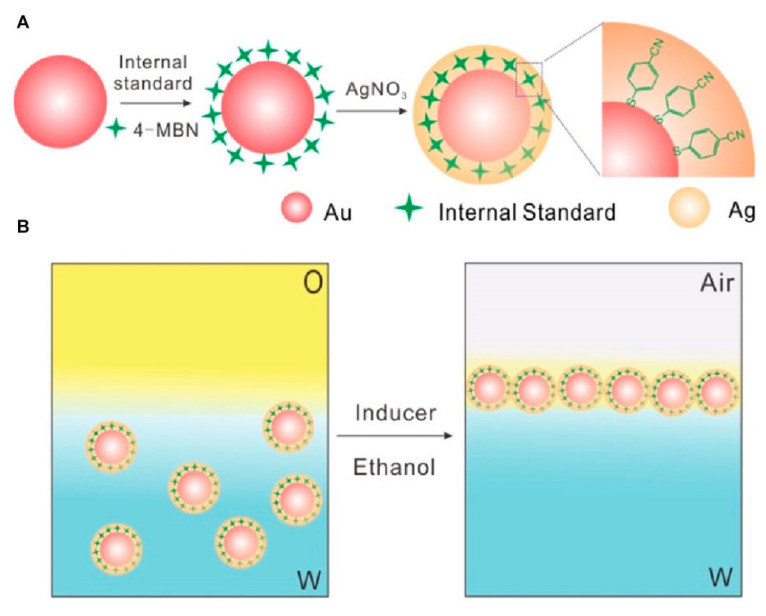
(**A**) Schematic illustration of the synthesis of Au@4-MBN@Ag NPs. (**B**) Illustration of self-assembly of Au@4-MBN@Ag NPs at the cyclohexane–H_2_O interface [125]. Reprinted with permission from Tian et al. Copyright 2024 Frontiers Media S.A.

Core–shell and hollow nanoparticles are promising building blocks for the construction of tunable and spectrally responsive 3D SERS substrates [122,123,124]. These architectures provide unique plasmonic advantages due to the confined electromagnetic (EM) fields at the shell–core interfaces and tunable optical properties. Typically, the self-assembly process involves two main steps: (i) solution-phase synthesis of core–shell nanostructures (e.g., Au@Ag, SiO_2_@Au) with well-defined morphology and shell thickness [125], as schematically illustrated in Figure 13A; and (ii) bottom-up assembly into ordered or quasi-ordered multilayer films via Langmuir–Blodgett (LB) deposition, drop-casting, or evaporation-induced self-assembly, as shown in Figure 13B.

One of the primary benefits of core–shell nanoparticles lies in their ability to concentrate and confine local EM fields at the core–shell boundary, especially when plasmonic metals such as Ag or Au are used as the shell. These localized hot spots significantly boost the SERS enhancement factor, particularly when assembled into densely packed arrays with controlled nanogaps. Additionally, core–shell structures enable precise tuning of the LSPR by adjusting the core diameter, shell thickness, and surrounding dielectric medium. This spectral tunability allows for optimization under specific laser excitation conditions to maximize Raman signal output. Moreover, core–shell systems provide enhanced structural stability and surface modifiability. For instance, SiO_2_@Au nanoparticles exhibit good dispersion stability and offer abundant surface functional groups for biomolecular conjugation. In contrast, Au@Ag nanoparticles combine the strong plasmonic activity of Ag with the chemical inertness and biocompatibility of Au, making them suitable for complex biological media.

Hollow nanoshells and nanocages represent another class of plasmonic nanostructures that support dual-surface enhancement (inner and outer shell). These structures introduce plasmonic cavity modes, which broaden the spectral range and introduce additional EM field confinement mechanisms. Multilayer assemblies of these particles can form 3D SERS films with high packing density, low interparticle variability, and excellent signal reproducibility. For example, Bernard et al. [126] fabricated SiO_2_@Au multilayer films via LB transfer, achieving EFs > 10^7^ and RSDs < 5% over large sensing areas. Furthermore, self-assembled core–shell structures can be integrated with functional elements, such as responsive polymer shells, magnetic cores, or molecular recognition moieties (e.g., antibodies, aptamers), to create multifunctional SERS platforms. These features allow for targeted detection, stimulus-controlled analyte release, or multiplexed biosensing.

Nevertheless, challenges remain in the uniformity of colloidal synthesis (e.g., shell thickness, size distribution) and environmental sensitivity during the film assembly process. To overcome these issues, recent advances have leveraged template-guided assembly, DNA-mediated positioning, and computational modeling of plasmonic coupling to enhance reproducibility and structural precision.

In summary, core–shell and hollow nanostructures offer a modular and scalable route to high-performance 3D SERS substrates, combining tunable plasmonic properties, dense hot spot formation, and functional versatility, making them highly suitable for biosensing, point-of-care diagnostics, and portable Raman detection platforms.

### 5.5. Hybrid Integration Strategies for Hierarchical Architectures

Hierarchical hybrid structures represent a more advanced class of SERS substrates. These architectures combine multiscaled structural domains and heterogeneous material compositions to synergistically integrate the advantages of diverse enhancement mechanisms, spatial arrangements, and functional modalities [127,128,129]. Compared to conventional mono-component systems, hybrid SERS substrates are purposefully engineered by assembling a variety of nanoscale building blocks (e.g., nanostars, porous frameworks, soft matrices, dielectric interfaces) through stepwise assembly, co-deposition, or multimodal crosslinking. The result is a composite material with enhanced plasmonic coupling, complex hot spot distributions, and environmental responsiveness.

On the mechanical scale, the rationale behind hybrid integration lies in leveraging plasmonic coupling modes and hierarchical architectures to intensify local electromagnetic fields at the nanoscale resolution. For instance, embedding sharp-tipped nanoparticles (e.g., Au nanostars) into aligned nanowire scaffolds enables the co-localization of hot spots due to vertical confinement and tip-induced field enhancement. Similarly, integrating plasmonic nanoparticles into responsive hydrogel matrices introduces dual functionalities: flexible analyte diffusion and optical light trapping via refractive index gradients. In some designs, dielectric or semiconductor materials are incorporated to facilitate charge-transfer-based chemical enhancement, thereby amplifying the SERS response.

**Figure 14:**
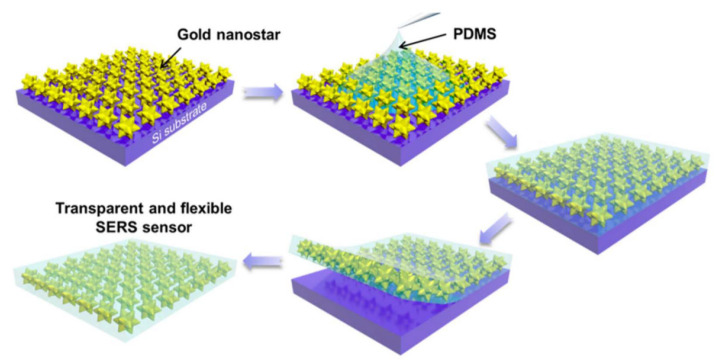
Schematic of hybrid integration for hierarchical SERS architectures [130]. Reprinted with permission from Park et al. Copyright 2017 American Chemical Society.

Recent studies have validated the potential of these hybrid platforms. For example, a transparent and flexible SERS sensor composed of gold nanostars embedded in a silicone rubber film has been reported [130] (see Figure 14). This sensor not only exhibits excellent optical transparency and mechanical flexibility but also enables sensitive detection of chemical and biological analytes on curved surfaces, highlighting the suitability of nanostructures for wearable sensing technologies. Furthermore, such dynamic systems offer promising solutions for developing reconfigurable or wearable SERS devices, where tunable responsiveness can enable real-time detection in fluctuating environments.

The fabrication of hybrid architectures typically involves a multi-step process: (i) synthesis of discrete nanocomponents with controlled morphology and surface chemistry, (ii) dispersion or immobilization of these components within a host matrix or scaffold, and (iii) stabilization of the final architecture through crosslinking, drying, or curing. To achieve spatially resolved sensing or microarray applications, emerging additive manufacturing techniques—such as inkjet or aerosol jet printing—have been adopted to deposit hybrid SERS materials onto curved or irregular surfaces.

However, several challenges remain with hybrid systems. Firstly, intercomponent compatibility—such as surface charge, dispersibility, and ligand stability—must be carefully managed to prevent aggregation or phase separation during processing. Secondly, structural and compositional heterogeneity often leads to poor batch-to-batch reproducibility. Additionally, the electromagnetic behavior in such complex hybrid environments remains difficult to model accurately; advanced simulation methods such as the finite-difference time-domain (FDTD) or finite element method (FEM) have limited predictive capabilities in these systems due to their inherent complexity. Despite these challenges, hierarchical hybrid integration offers unmatched design freedom and multifunctionality. These systems are especially attractive for real-world SERS applications, where a combination of structural flexibility, chemical selectivity, and mechanical robustness is essential. Moreover, their compatibility with wearable electronics, microfluidics, and lab-on-a-chip technologies opens up new avenues in point-of-care diagnostics, on-body sensing, and environmental monitoring.

### 5.6. Comparative Evaluation of Fabrication Methods

While Section 5.1, Section 5.2, Section 5.3, Section 5.4 and Section 5.5 have outlined the key fabrication strategies for constructing 3D SERS substrates, a comparative evaluation is essential to guide practical selection. Different fabrication methods offer unique strengths and limitations in terms of structural uniformity, hot spot distribution, reproducibility, cost, and compatibility with biosensing applications. Table 3 summarizes these methods based on key performance metrics and structural considerations reported in the literature.

This comparative analysis highlights that template-assisted and self-assembly techniques are well-suited for large-area reproducible fabrication, while electrochemical and dealloying-based methods offer simplicity and high enhancement at the expense of structural control. Hybrid fabrication, though powerful for multifunctional sensing, may involve more complex processing and quality control. Therefore, the choice of fabrication strategy should be tailored to the intended application—whether the priority is the enhancement factor, reproducibility, flexibility, or integration with biological systems.

## 6. Biosensing Applications in Biological Sciences

SERS technology has proven to be one of the most useful analytical technologies during the past few decades, and the mechanisms behind the technology, as well as the substrate engineering, have been analyzed and refined in an extreme level of detail. During the last several years, SERS biosensors have found wide application in in vivo and in vitro sensing. In this section, we shall summarize advancements and applications of SERS biosensors in the biological sciences.

### 6.1. The Biocompatibility of SERS Substrate Materials

The application of SERS substrates in biological and biomedical fields, such as biosensing, bioimaging, and drug delivery, has been a growing area of research. However, the practical applications of these platforms, particularly for in vivo sensing, critically depend on their biocompatibility. Therefore, it is necessary to consider the biocompatibility and toxicity of the SERS substrate materials. Chitosan holds excellent biocompatibility, biodegradation, and a high affinity for anionic biomolecules. Childs and coworkers fabricated chitosan-coated Au/Ag nanostars as a SERS probe for protein detection [131]. Chowdhury and colleagues developed a biofunctionalized 3D carbon nanonetwork platform, which possessed great biocompatibility and cell adhesion capacity [132]. This team firstly used these carbon-based materials as the nonplasmonic SERS substrate material for in vitro detection and differentiation of HeLa cells and fibroblasts [133]. Yang and coworkers designed a two-photon sensing platform for live-tissue bioimaging and systematically evaluated its biocompatibility using an MTS assay in contact with HeLa cells [134]. Remarkably, cell viability remained above 90% even at a high probe concentration, confirming the platform’s excellent biocompatibility. Given the critical role of cytocompatibility in biocompatibility, Chen et al. engineered a robust bimetallic Mo-Ag film that exhibited good cytocompatibility with MC3T3-E1 cells [135]. This robust performance under biologically relevant conditions highlights the potential for SERS detection in vivo. In addition, a polymer has been used to functionalize SERS substrate materials to enhance the stability and biocompatibility and increase the cellular internalization efficiency. For instance, Zhang and colleagues developed a pH-responsive plasmonic nanosensor (PA-coated SERS probe) as the plasmonic substrate, and the biocompatibility of this platform was investigated in the MCF7 breast cancer cell line [136]. The study found that SERS probes coated with PA and those coated with PEG showed comparable cell viabilities across concentrations ranging from 2 to 50 μg/mL. Even at the highest concentration tested, cell viability remained above 85%. Schumacher and colleagues developed functional gold nanoparticles (AuNPs) for in vivo bioimaging, utilizing poly(isoprene)-diethylenetriamine (PI-DETA) as the nanoparticle ligand and poly(isoprene)-*block*-poly(ethylene glycol) (PI-*b*-PEG) as a biocompatible micelle-forming agent. The quantity of co-encapsulated AuNPs was controlled by adjusting the PI-*b*-PEG-to-PI-DETA ratio during solvent-driven self-assembly [137].

### 6.2. Glucose Sensing

Raman spectrometry is a powerful molecule detection instrument since the method provides clear spectral fingerprints of each analyte. However, its poor sensitivity has long limited its application in trace chemical analysis. This weakness was addressed through the invention of SERS in the 1970s, when it became possible to transform the field of ultrasensitive detection [138,139]. Despite that, glucose sensing using SERS has been observed to be challenging because of the low Raman scattering cross-section of glucose and low adsorption of glucose on the surfaces of metal nanoparticles. To overcome these challenges, researchers have been interested in attempting to develop a functionalized SERS substrate to detect glucose precisely. To give an example, Sun and colleagues [140] produced an aligned silver nanorod substrate functionalized with 4-mercaptophenylboronic acid (4-MPBA), where under glucose binding to the boronic acid functional group, the SERS signal changed proportionally to the glucose concentration. Similarly, Raju et al. [141] prepared a glucose-sensing platform system using silver nanocluster films modified with 2-thienylboronic acid (2-TBA), which served as a molecular bridge between the silver surface and the glucose molecules. The system enabled a quantitative measurement by a characteristic Raman band at 986 cm^−1^, and the system had a linear response over the clinically relevant concentration range (1–500 μM). This is very remarkable because it can potentially offer noninvasive glucose measurement in the treatment of diabetes, particularly through saliva. The further evolution of the field was marked by the work by Jiang and co-authors, who became the first to develop a label-free glucose sensor based on the application of the self-assembled Ag NPs of varying size, with the detection limit of 6.62 pM [142]. All these studies indicate that the SERS-based platforms that use Ag NPs as the substrate materials can be utilized as sensitive and selective glucose detectors.

The use of gold nanoparticles (Au NPs) as a multipurpose bioassay platform is possible because of their intrinsic plasmonic property and enzyme-mimicking activities. Israa et al. [143] have prepared a label-free SERS biosensor using a Au@silica core–shell nanoparticle with a glucose oxidase (GOx) enzyme decorated on the surface. In this system, glucose was oxidized by the GOx enzyme, and H_2_O_2_ was formed near the surface of the nanoparticles. The localized H_2_O_2_ has a significantly stronger SERS signal on laser excitation that is linearly proportional to the concentration of glucose so that it can be quantitatively detected. Hu and colleagues [144] took advantage of the innate peroxidase-like capacity of Au NPs to develop a peroxidase-like nanozyme through encapsulation of Au NPs in a highly porous and thermo-resistant metal–organic framework (Au NPs@MIL-101). The simultaneous glucose and lactate were quantified by GOx functionalization and lactate oxidase (LOx) in SERS. The multiplexed platform has demonstrated very promising outcomes in both in vitro and in vivo tests, and this means that it can be applied in clinical diagnostics.

The last few decades have been devoted to the elaboration of noninvasive glucose detection methods. Included among them was the fabrication of a plasmonic core–shell nanosensor (Au-PATA-Ag) to detect urinary glucose, which was found to have a high degree of specificity and stability in physiological conditions, which means great applicability in practice [145]. Innovative materials, such as composite materials, have also enhanced SERS performance. Hu et al. hybridized Au NPs with a porphyrin-based metal–organic framework, and consequently, an enzyme-free tandem reaction system was constructed with a higher sensitivity on the basis of the plasmonic resonance [146]. In other research, Man and colleagues have fabricated a MoS_2_ spaced Au-Ag bimetallic composite SERS-SPR sensor [147]. This sensor showed a sensitivity of 2473.37 nm/RIU, which is two times more sensitive than conventional monometallic systems (e.g., Ag-based SRP sensor: 1144.07407 nm/RIU [148]), and it was able to detect glucose linearly within a range of 0.625–20%. New materials such as MXene have also been promising. Cui et al. prepared a highly sensitive, reproducible, and stable Au NPs@MXene SERS substrate [149]. The SERS biosensor using Au@MXene can be created as a quantitative tear glucose sensor with the concentration range of 1 to 50 uM and LOD of 0.39 uM. To be used as wearable, Wang and colleagues designed an ultra-thin layered flexible SF-AAO-Au substrate that can perform simultaneous sweat glucose detection (10^−7^–10^−3^ M, LOD 1.68 × 10^−7^ M) and pesticide monitoring (LOD~5.7 ppt) to solve the issue of multiplexed sensing [150]. Hydrogel microsphere-based SERS platforms are fabricated to simplify the analysis of biological samples by pretreatment-free detection of glucose with high sensitivity and reproducibility as a new potential diagnostic method of diabetes [151]. Moreover, wearable SERS-based sensors hold excellent potential for offering detailed health insights by monitoring glucose present in body fluid. For example, Atta et al. designed an Au nanostar-based SERS substrate that can concurrently detect three sweat biomarkers—lactate, urea, and glucose—exhibiting LODs of 0.7, 0.6, and 0.7 μM, respectively. These LODs are notably lower than the clinically relevant concentrations of these biomarkers in sweat [152]. In that year, Yuan and coworkers reported a self-adhesive, biocompatible, and wearable microfluidic chip for glucose sensing [153]. The design incorporated erasable and reproducible plasmonic hot spots within the microfluidic channels, and the SERS substrate was functionalized with 4-mercaptophenylboronic acid to achieve exceptional sensitivity, reaching a remarkable LOD of 1 ng/L for glucose. In summary, wearable SRES-based biosensors demonstrate significant potential for noninvasive, real-time monitoring of glucose.

### 6.3. Tumor Sensing

Today, cancer has been acknowledged as a heterogeneous collection of diseases that must be screened differently, and various subtypes of cancer have different tumor biomarkers. SERS has in recent years been employed extensively in biomarker identification because of its superiority over conventional diagnostic methods. Reza and colleagues suggested a multiplexed SERS platform integrating shear forces induced by alternating current electrohydrodynamic and SERS nanotags to simultaneously detect the presence of protein biomarkers (HER2, MUC1, EGFR, and MUC16) overexpressed in breast, lung, and ovarian cancers [154]. This platform proved to be reliable and better than single-biomarker analysis, and it portrayed potential aspects of point-of-care usage. Based on this, Chen et al. developed a multiplex vertical flow SERS assay to detect different biomarkers of prostate cancer (prostate-specific antigen (PSA), carcinoembryonic antigen (CEA), and alpha-fetoprotein (AFP)) simultaneously in a single test spot [155]. The resulting biosensor has detection limits of 0.37, 0.43, and 0.26 pg/mL of PSA, CEA, and AFP, respectively, and is a good point-of-care diagnosis candidate. The engineering of nanostructures has also enhanced SERS uses. Au nanodot-encapsulated hollow mesoporous nanostructures were developed by Liu et al., and these can be applied as SERS probes to differentiate between cancer cells in vitro by measuring the amount of H_2_O_2_ [156]. This SERS probe was enzyme-conjugated and used in the detection of the respective substrates of glucose and uric acid. The effective synthesis of this SERS probe gives many other metallic nanodot-loaded hollow mesoporous nanostructures, which have a lot of applications in medical biology. Also, graphene oxide (GO) has been effectively utilized as an Au NP-modified SERS substrate. Au NPs@GO substrates enabled the detection of carcinoembryonic antigen at the ultratrace level (LOD ~ 12.5 fg/mL) by Basu et al. [157]. With the development of a wearable SERS technique and artificial intelligence (AI), researchers have integrated these techniques to develop a novel and precise method for cancer diagnosis. For instance, Chen et al. developed a hydrogel-based, flexible, wearable sweat sensor for SERS-AI monitoring of the treatment effect of lung cancer [158]. AI algorithms were used to identify characteristic biomarkers of lung cancer associated with various comorbidities. This wearable sweat sensor can successfully diagnose three treatment effects (progressive disease, partial response, and no change) with an accuracy of 89.7%, and they hold significant potential for clinical application.

Proteins as large biomolecules perform various biological functions, including metabolic reactions, immunity, transportation, and signal transduction [159]. Therefore, proteins are often regarded as the biomarkers of diseases, and detection of proteins is increasingly important for early diagnosis and treatment [160]. Nowadays, liquid chromatograph-mass spectrometry, nuclear magnetic resonance, and other protein detection methods have been used for the clinic diagnosis of diseases. However, these methods are time-consuming and costly. In recent years, SERS, as a new technology for protein detection, has gained widespread popularity. Even SERS has been used in the identification and detection of proteins, but there is always a limit to the use of SERS due to low Raman cross-sections of proteins. In order to solve this problem, nucleic acid–peptide conjugates have been created, which have amazing potential in sensing and conjugation of aptamers and allow better biometrics and programmability. To illustrate this, Su et al. developed an aptamer–peptide SERS probe to evaluate the aberrant protein levels of cancer patients directly and in high specificity [161]. It is worth noting that following the analysis of cryopreserved blood samples using this SERS platform, the outcomes showed that the platform gave false negative outcomes when the samples were taken out at 4 °C after more than 2 days. Oliveira et al. fabricated an Au nanostar-based SERS biosensor for point-of care detection of the cancer biomarker CA 15-3 [162]. The biosensor was fabricated by depositing molecularly imprinted polymers (MIPs) on a gold electrode, followed by conjugation with antibody-labeled Au nanostars loaded with a suitable Raman reporter for signal enhancement. A strong Raman peak at 1079 cm^−1^ was observed, and its intensity increased proportionally with CA 15-3 concentrations (0.016–248.51 U/mL), indicating higher probes binding at elevated biomarker levels. Microwave ablation (MWA) is widely used in cancer treatment. The lack of indicators to assess the thoroughness of the ablation process has limited the application of MWA. To address this limitation, Ouyang et al. reported a novel SERS-based strategy to identify serum biomarkers for evaluation of the completeness of tumor ablation [163]. The platform showed a linear detection range of 0.1 pg/mL to 1 ng/mL, with LODs of 0.082 pg/mL for CCL20 and 0.096 ng/mL for EGF. This strategy supplied a promising tool for improving the assessment of MWA efficacy, ultimately contributing to better clinical outcomes and patient survival rates.

Exosomes released by cancer cells have been under focus as a target for the early detection of cancer because they are used in cell-to-cell communication and they contain disease-specific biomarkers. The development of the SERS technique in the recent past has made sensitive and specific analysis of exosomes possible. Chen et al. have synthesized 4-MPBA-labeled Ag NPs, which are able to bind on the surface of exosomes and acquire SERS-active hybridized exosomes [164]. The outcomes showed that this detection platform displayed a dual functionality, as it could be used in intracellular imaging and simultaneous pH detection (range 3.0 to 8.0) via SERS signals. Due to the ability of microRNAs (miRNAs) in exosomes to ensure the effective regulation of gene expression, they can be applied as potential biomarkers of cancer diagnosis. Kang et al. developed a SERS-based sensor with a highly dense and ordered Au octahedral structure to detect let-7a miRNAs in MCF-7 exosomes quantitatively [165]. The sensor had a wide linear range (10 aM–10 nM) and an ultrasensitive detection limit (5.3 Am) with no signal amplification, as well as detecting drug-induced shifts in miRNA expression. Not only could this SERS-based sensor accurately detect the presence of the let-7a miRNAs but also it could be used to assess the impact of drug treatment on the exosome expression of the let-7a miRNAs. Still, there are limitations to the detection of ultra-trace concentrations of exosomes in a complex biological sample. This was resolved by Gu et al., who developed a new SERS aptasensor for gastric cancer exosomes [166]. This SERS aptasensor developed was very helpful as a reference in the clinical detection of early cancer due to its fast response, high sensitivity and specificity, good repeatability, and good recoveries. Based on this, recently, the same team has constructed an ultrasensitive SERS aptasensor by integrating branched hybridization chain reaction and a tetrahedral DNA-based trivalent aptamer to determine cancer-derived exosomes with great specificity [167]. The response range of this sensing platform was remarkable (1.4410^3^–1.4410^7^ particles/mL) and its sensitivity (LOD~390 particles/mL) was unprecedented, facilitating the detection of a single exosome in 2 μL samples. Pang et al. designed a dual-SERS biosensor assisted by duplex-specific nuclease (DSN) to quantify miRNA-10b in exosomes and residual plasma of blood samples, using the Raman peak intensity of DTNB at 1332 cm^−1^ as the detection signal, achieving an ultralow LOD of 1 aM [168]. Similarly, Tang et al. developed an Au–Ag mushroom-shaped SERS probe combined with exponential amplification reaction (EXPAR) to detect exosomal miR-375, a biomarker for prostate cancer, with a detection limit of 10 fM [169]. This approach effectively distinguishes serum samples from healthy donors and prostate cancer patients, demonstrating significant potential for clinical diagnosis of prostate cancer. All of these studies demonstrate the potential of exosome-based SERS platforms in early cancer diagnosis and have the benefits of high sensitivity, multiplexing ability, and clinical utility.

### 6.4. Drug Delivery

Au NPs and Ag NPs have become the ideal platforms of drug delivery systems (DDSs) due to their special plasmonic features and adjustable surface features. They have a clear size and controllable shapes that allow the exact engineering of the process and therapeutic use. Notably, the targeted accumulation of their nanoparticles in the tumor tissues occurs due to the enhanced permeability and retention (EPR) effect in tumor tissues, and the overall exposure to the nanoparticles is reduced. As an example, Shao et al. designed a novel Janus silver–mesoporous silica nanocarrier (Ag-MSN), which can integrate SERS traceability and drug delivery [170]. The composite structure takes the advantage of the high SPR of Ag NPs and the high surface area of mesoporous silica, and it attains both improved SERS imaging and an increased drug-loading capacity. These nanocarriers may be loaded with doxorubicin (DOX) to preserve the SERS signal, allow targetable detection and traceable drug delivery. Moreover, the Ag-MSNs loaded with DOX also had great destructive effects on cancer cells but caused less damage to normal cells, and DOX-loaded Ag-MSNs had the potential of reducing DOX-induced damage to normal tissue cells. In 2023, Chen and colleagues prepared a MXene/Ag NP film that enabled SERS-traceable drug delivery [171]. This film was loaded with anticancer drug DOX via 4-MPBA, not only as linker molecule but also as probe molecule to be detected by following SERS. They considered GSH to be a more useful release cue than cysteine, indicating the role of stimulus-responsive design in the development of nanocarriers.

Au NPs have outstanding biocompatibility and surface modification properties to deliver drugs, but they have weak intrinsic Raman signals to be tracked precisely. To address this shortcoming, anisotropic Au nanostructures such as sharp-ending metal surfaces (e.g., nanostars, nanoflowers) have been designed as SERS amplifiers. As an example, Tian and colleagues prepared PEGylated Au nanostars conjugated to mitoxantrone (MTX nanostars) in HEPES-buffered seed-mediated synthesis [172]. The literature shows that MTX nanostars are able to quench the majority of the fluorescence of organic molecules in the presence of a near-infrared excitation source. Moreover, the signal of MTX nanostars was enhanced within the cells compared to outside of the cells, resulting from the fact that the Au nanostar-containing cells served as a focusing medium and the maximum enhancement occurred within the cells. The conclusions indicated that MTX nanostars have the potential to be candidates for intracellular SERS detection and drug delivery through Raman/FTIR imaging. Another breakthrough in this paradigm was made by Song et al., who designed multifunctional gold nanoflowers to enable intracellular SERS imaging-guided chemo-phototherapy [173]. Raman and RGD molecules were used to modify the gold nanoflowers (AuNFs), to give the AuNFs traceable and targeted properties. Then, thiolated-PAA was prepared and used to coat AuNFs with a layer of negative charges to enable pH-dependent loading and releasing of DOX efficiently. As RGD has the ability to specifically target the overexpressed 2v3 in A549 cell, the AuNF SERS tags obtained can show clear SERS signals upon incubation with A549 cells. The findings showed that the AuNF SERS tags are effective with tracing and targeted imaging.

The traditional anticancer treatments are usually characterized by systemic toxicity and quick resistance. In an attempt to overcome these shortcomings, DDS nanoparticles have been formulated with the aim of maximizing therapeutic effects and reducing the side effects. Nevertheless, it is difficult to monitor the release of drugs in real time. Photobleaching, cellular autofluorescence, and spectral overlap in multiplexed detection are limitations of fluorescence-based tracking, a strategy that is nevertheless in widespread use [174]. Conversely, SERS is more sensitive, photostable, and multiplexable, and thus a perfect instrument to monitor drug delivery at the single-cell level. As an example, Liu and colleagues prepared a pH-responsive DDS that was composed of a silica nanoparticle scaffold with SERS-active Au NPs attached [175]. DOX was loaded in this DDS and could only be released in the low-pH environment of the tumor and not in physiological conditions. SERS mapping and fluorescence assays on HeLa cells revealed that SERS had a better spatial resolution and greater details of intracellular drug distribution patterns. The next step in the real-time monitoring was the development of an integrated SERS platform by Manago and his colleagues to deliver drugs and monitor Galunisertib release qualitatively in live cancer cells simultaneously [176]. This system was sub-femtogram sensitive, which can provide the label-free detection of drug release kinetics. The SERS was validated using high-performance liquid chromatography (HPLC) and was found to be a highly sensitive and reliable substitute to the conventional analysis methods. Protein coronae have in recent years been used to decorate the surface of nanoparticles, to construct a multiple functional platform of nanomedicine. As an example, Hong and co-workers synthesized nanoparticles composed of poly(ε-caprolactone) (PεCL) cores, Au NP shells, and BSA coronae via a self-assembly technique [177]. The hydrophobic core was employed to encapsulate curcumin, a Au NP shell with a Raman probe can serve as a SERS substrate, and the protein coronae was functionalized with folic acid to target 4T1 cells. This design not only improved drug loading and tumor targeting but also enabled sensitive SERS detection of cellular uptake, demonstrating the potential of multifunctional SERS-DDS hybrids for precision medicine.

Bimetallic nanostructures have been demonstrated to have a positive synergistic effect. A Au@Ag complex performs better than single metal systems due to improved electronic and catalytic interactions [178]. The recent past has seen the development of Au@Ag complexes with multifunctional nanomaterials, which have enhanced the biomedical application of the Au@Ag complexes, particularly in targeted drug delivery and real-time monitoring. In a case example, Chen et al. proposed a dual-targeting SERS-encoded graphene oxide (GO) nanocarrier to perform co-delivery of anticancer drugs [179]. GO in this system was anchored to Au@Ag nanoparticles and Fe_3_O_4_ nanoparticles, which provided the composite with a high SERS activity and magnetic targeting ability. The nanocarriers formed could be internalized receptor-mediated into cancer cells, and localized in lysosomes, which was confirmed by fluorescence-SERS dual-modal tracking. Here, 9-aminoacridine and DOX could be co-delivered and the dynamic distribution of the drugs followed using spectral techniques. Further, to enhance multifunction, Huang and co-workers synthesized an Fe_3_O_4_ @Au@Ag hybrid nanoplatform based on GO to incorporate drug loading, stimuli-responsive release, real-time SERS, magnetic resonance imaging, and photothermal therapy [180]. The Fe_3_O_4_ @Au@Ag nanoparticles could be stabilized by a GO substrate and the strong SERS signal could be preserved, which ensured a high reproducibility and stability. It is noteworthy that this system could allow the release of DOX in vitro and in vivo, indicating that the system can be useful in monitoring the release of optically inactive therapeutic agents. Nevertheless, despite the progress, the conventional blood sampling remains the gold standard when speaking of pharmacokinetic analysis, but is restricted by its invasiveness and applicability to point-of-care treatment. Li et al. have addressed this challenge by fabricating pain-free, SERS-active microneedles that allow them to monitor the drugs in real time in the dermal interstitial fluid [181]. The microneedles exhibited sensitive quantitation of methylene blue in a murine model 10 min following injection, and the interstitial fluid concentration of the drug was highly correlated with the plasma concentration. The interstitial fluid concentration of mitoxantrone was, however, 2–3 orders lower than the blood concentration, hence why a particular calibration of the compound was necessary. It is noninvasive and could offer an alternative to blood-based analysis, especially in dermal drug delivery systems.

### 6.5. Advantages in 3D SERS in Detection of Low-Abundance Biomarkers

Three-dimensional SERS has emerged as a powerful tool for the detection of ultralow-abundance biomarkers, addressing critical limitations in conventional diagnostic methods. Unlike fluorescence or enzyme-linked immunosorbent assays (ELISAs), SERS amplifies Raman signals by 10 to 14 orders of magnitude through localized surface plasmon resonance generated by abundant hot spots [182]. Recent advances in SERS techniques have significantly enhanced its capability for early diagnosis through ultrasensitive biomarker detection. A representative example is the work by Wang et al., who engineered an ultrasensitive SERS sandwich biosensor for bacterial detection [183]. This platform integrated two key components: (1) the bioinspired signal module employing a dendritic mesoporous silica nanocarrier (DMSN) loaded with plasmonic nanoparticles and a SERS tag, and (2) the plasmonic enrichment module based on Au-coated magnetic Fe_3_O_4_ nanoparticles. The DMSN can effectively shrink nanogaps between plasmonic nanoparticles to improve hot spot intensity, while the plasmonic enrichment module can supply more hot spots. Remarkably, this sensor achieved ultrahigh sensitivity (7 CFU/mL) and demonstrated clinical potential through in vivo experiments, enabling rapid and precise bacterial detection in serum samples from septic mice—a critical advancement for early diagnosis of bacterial sepsis. The well-established correlation between exosomal miRNA expression profiles and cancers has driven the development of advanced SERS-based biosensors with broad linear response ranges and ultralow detection limits. Lee and coworkers have fabricated a SERS-based sensing platform for multiplex detection of breast cancer-specific miRNAs (miR-21, miR-222, and miR-200c) [184]. The platform showed concentration-dependent SERS signal enhancement, demonstrating linear responses across a broad dynamic range (1 aM to 100 nM) with an impressive LOD of 1 aM. Compared with previous studies, the LOD of this platform was at least 100-fold lower than other methods using several amplification reactions. Moreover, Zhang et al. have designed a novel cascade signal amplification strategy for ultrasensitive detection of gastric cancer-associated miRNA-106a using the SERS technique [185]. This approach achieved exceptional sensitivity with a LOD of 8.55 aM, demonstrating a linear response across a wide concentration range (10 aM to 1 nM) while maintaining high specificity. Recently, the SERS technique has been employed as the ultrasensitive tool for the quantification of multiple Alzheimer’s disease (AD) biomarkers. For instance, Zhan et al. proposed a SERS-lateral flow assay (SERS-LFA) for simultaneous detection of multiple AD biomarkers (Amyloid-beta 42, Amyloid-beta 40, tau proteins, and neurofilament light chain) [186]. The LODs of these four biomarkers reached the fM level, and they were two orders of magnitude lower than the concentration in blood. The plasma phosphorylation of tau at the Ser (396, 404) (p-tau^396,404^) level appears to be a promising biomarker of AD. Zhang and coworkers designed a colorimetric and SERS-LFA platform for rapid, highly sensitive and robust detection of plasma p-tau^396,404^ [187]. This platform realized an LOD of 3.8 pg/mL by SERS without cross-reacting with other tau species. Furthermore, the team developed a colorimetric and SERS dual-mode immunosensor capable of detecting p-tau^396,404^ in the blood with an LOD as low as 1.5 pg/mL in SERS mode and 24 pg/mL in colorimetric (mode visible to the naked eye) [188]. These ultrasensitive platforms can offer powerful tools for the clinical diagnosis of early diseases.

## 7. Conclusions and Outlook

In summary, 3D SERS substrates offer significant advances over traditional two-dimensional (2D) systems by increasing hot spot density, enhancing light–matter interaction through multiple scattering, and enabling more robust biosensing architectures. Through diverse structural types—including vertically aligned nanowires, dendritic networks, porous frameworks, core–shell assemblies, and hierarchical hybrids—3D SERS platforms have demonstrated outstanding enhancement factors (EFs), reproducibility, and flexibility in design. Progress in fabrication methods such as template-assisted growth, dealloying, and self-assembly has made it possible to tailor structural parameters for optimized SERS activity. These advances have expanded the application of SERS in biological fields, including glucose sensing, tumor marker detection, and real-time monitoring of physiological parameters.

However, despite these achievements, several critical challenges remain that must be addressed before 3D SERS can be widely applied in real-world biological and clinical environments:

(1) Stability in Complex Biological Matrices: Biological fluids such as blood, saliva, or tissue homogenates are chemically complex and prone to non-specific adsorption of proteins and macromolecules onto nanostructured surfaces. This can lead to degradation of the signal intensity or aggregation of the nanostructures, particularly in silver-based substrates. Core–shell designs (e.g., Au@Ag) and surface passivation strategies (e.g., PEGylation) are promising, but more universal stabilization methods are needed for long-term operation in variable physiological conditions.
(2) Signal Attenuation Under Extreme or Variable Conditions: Environmental stressors such as high ionic strength, fluctuating pH, temperature changes, and oxidative conditions can impair signal reproducibility. Substrates embedded in hydrogels or flexible matrices may undergo deformation or swelling, disrupting the hot spot architecture. Therefore, further work is required to engineer mechanically and chemically resilient substrates that can retain signal fidelity under such stresses.
(3) Lack of Standardization and Reproducibility: Many 3D SERS fabrication strategies, especially those based on electrochemical growth or dendritic self-assembly, still suffer from high batch-to-batch variability. This hinders quantitative biosensing applications and large-scale deployment. Introducing scalable, template-guided synthesis and machine learning-guided optimization may help overcome this limitation.
(4) Optical Interference and Quantification in Turbid Media: Signal attenuation due to scattering, absorption, or matrix interferences in turbid samples remains a major issue for in vivo and real-time applications. Integration of optical trapping designs, internal standards, and ratiometric SERS techniques can enhance spectral reliability, but requires further refinement for clinical use.

In addition to these scientific and technical barriers, broader commercialization of 3D SERS substrates also faces several practical challenges. Fabrication techniques that offer excellent sensitivity are often complex, low-throughput, and cost-inefficient, hindering industrial scalability. Moreover, mechanically fragile architectures such as dendritic networks and aerogels are difficult to integrate into portable or wearable biosensing devices. Regulatory concerns related to reproducibility, long-term stability in biological fluids, and biocompatibility further constrain clinical adoption. To overcome these issues, future research should focus on scalable manufacturing approaches—such as roll-to-roll printing, self-assembly under mild conditions, or 3D nanoimprinting—and the use of robust, flexible, or composite substrate materials. Additionally, standardization of SERS performance metrics and integration with machine learning for design optimization and quality control will be essential to translate 3D SERS from the laboratory into commercial products.

Looking forward, the rational design of multifunctional, stimuli-responsive, and biocompatible 3D SERS substrates—coupled with advanced computational modeling and real-time signal processing—holds great promise for overcoming current limitations. Achieving a reliable performance in complex environments will be key to realizing the full potential of 3D SERS in practical biomedical diagnostics, environmental monitoring, and point-of-care testing.

## Figures and Tables

**Table 1:** Performance comparison between 2D and 3D SERS substrates.

Feature	2D SERS Substrates	3D SERS Substrates
Hot Spot Dimension	Confined to planar surface	Distributed volumetrically in all dimensions
Enhancement Factor	10^5^–10^7^	>10^8^
Reproducibility	Moderate	High (RSD typically < 10%)
Analyte Accessibility	Limited diffusion on surface	Enhanced diffusion via pores and 3D networks
Fabrication Methods	Lithography, self-assembly, etc.	Template growth, dealloying, freeze-drying, etc.

**Table 2:** Summary of key characteristics of different 3D SERS substrate architectures.

Architecture Type	Typical EF	Reproducibility (RSD%)	Structural Features	Advantages
Nanowires/Nanorods	10^7^–10^8^ [27,28]	≤8%	High aspect ratio; vertical light confinement	Uniform hot spot; scalable fabrication; strong EM
Dendritic/Fractal Nanostructures	>10^8^ [25,26]	5–15%	Multibranched; high-curvature tips; fractal geometry; electrochemically grown	Ultrahigh enhancement; broadband plasmonic response
Porous Frameworks/Aerogels	10^7^–10^9^ [29,30]	≤10%	Interconnected pores/ligaments; high surface area; tunable porosity	Efficient light trapping; large sensing volume; fast analyte diffusion
Core–Shell and Hollow NPs	10^6^–10^8^ [32,83]	≤5%	Tunable core–shell interfaces; shell thickness control; cavity modes	High reproducibility; spectral tunability; suitable for multilayer assembly
Hierarchical Hybrid Structures	>10^9^ [101]	≤10%	Multiscale integration	Flexibility; responsiveness; enhanced hot spot density

**Table 3:** Summary of fabrication methods for 3D SERS substrates and their comparative advantages.

Fabrication Method	Key Features	Advantages	Limitations	EF/RSD
Template-Assisted Growth	Uses AAO, colloidal crystals, bio-templates	High uniformity; tunable geometry; good reproducibility;	Limited design freedom; template removal steps; sometimes low throughput	EF > 10^7^; RSD < 7%
Electrochemical Dendrite Growth	Self-formed fractal structures on electrodes	simple equipment; broadband EM enhancement; scalable to large areas	Poor reproducibility; random morphology; fragile structure	EF > 10^8^; RSD ~ 15%
Dealloying/Freeze-Drying	Nanoporous metals; aerogels from sol–gel or metal–organic systems	High surface area; excellent light trapping; fast analyte access	Brittle (aerogels); pore size hard to control; waste generation	EF > 10^7^~10^8^; RSD < 10%
Colloidal Self-Assembly	Langmuir–Blodgett, evaporation-driven, drop-casting of particles	Cost-effective; scalable; tunable interparticle gaps	Sensitive to humidity and solvent; requires precise shell thickness control	EF > 10^7^; RSD < 5%
Hybrid/Hierarchical Integration	Combines nanowires, nanostars, hydrogels, photonic crystals	Multifunctional sensing; responsive materials; enhanced light localization via hierarchical design	Structural complexity; reproducibility challenges; difficult to model and fabricate at scale	EF > 10^9^ RSD varies
